# Large-scale discovery of novel neurodevelopmental disorder-related genes through a unified analysis of single-nucleotide and copy number variants

**DOI:** 10.1186/s13073-022-01042-w

**Published:** 2022-04-26

**Authors:** Kohei Hamanaka, Noriko Miyake, Takeshi Mizuguchi, Satoko Miyatake, Yuri Uchiyama, Naomi Tsuchida, Futoshi Sekiguchi, Satomi Mitsuhashi, Yoshinori Tsurusaki, Mitsuko Nakashima, Hirotomo Saitsu, Kohei Yamada, Masamune Sakamoto, Hiromi Fukuda, Sachiko Ohori, Ken Saida, Toshiyuki Itai, Yoshiteru Azuma, Eriko Koshimizu, Atsushi Fujita, Biray Erturk, Yoko Hiraki, Gaik-Siew Ch’ng, Mitsuhiro Kato, Nobuhiko Okamoto, Atsushi Takata, Naomichi Matsumoto

**Affiliations:** 1 Department of Human Genetics, Yokohama City University Graduate School of Medicine, Yokohama, Japan; 2 Clinical Genetics Department, Yokohama City University Hospital, Yokohama, Japan; 3 Department of Rare Disease Genomics, Yokohama City University Hospital, Yokohama, Japan; 4 Faculty of Nutritional Science, Sagami Women’s University, Sagamihara, Japan; 5 Department of Biochemistry, Hamamatsu University School of Medicine, Hamamatsu, Japan; 6 Department of Pediatrics, Aichi Medical University, Nagakute, Japan; 7 Department of Medical Genetics, Ege University Faculty of Medicine, Izmir, Turkey; 8 Current affiliation: Department of Medical Genetics, Prof. Dr. Cemil Tascioglu City Hospital, Istanbul, Turkey; 9 Hiroshima Municipal Center for Child Health and Development, Hiroshima, Japan; 10 Department of Genetics, Penang Hospital, Penang, Malaysia; 11 Department of Pediatrics, Showa University School of Medicine, Tokyo, Japan; 12 Department of Medical Genetics, Osaka Women’s and Children’s Hospital, Izumi, Japan; 13 Laboratory for Molecular Pathology of Psychiatric Disorders, RIKEN Center for Brain Science, Wako, Japan

**Keywords:** Neurodevelopmental disorder, Intellectual disability, Epileptic encephalopathy, Autism spectrum disorder, Rare disease, De novo variant, Copy number variant, Copy number variation, Mutation rate, Deep learning

## Abstract

**Background:**

Previous large-scale studies of de novo variants identified a number of genes associated with neurodevelopmental disorders (NDDs); however, it was also predicted that many NDD-associated genes await discovery. Such genes can be discovered by integrating copy number variants (CNVs), which have not been fully considered in previous studies, and increasing the sample size.

**Methods:**

We first constructed a model estimating the rates of de novo CNVs per gene from several factors such as gene length and number of exons. Second, we compiled a comprehensive list of de novo single-nucleotide variants (SNVs) in 41,165 individuals and de novo CNVs in 3675 individuals with NDDs by aggregating our own and publicly available datasets, including denovo-db and the Deciphering Developmental Disorders study data. Third, summing up the de novo CNV rates that we estimated and SNV rates previously established, gene-based enrichment of de novo deleterious SNVs and CNVs were assessed in the 41,165 cases. Significantly enriched genes were further prioritized according to their similarity to known NDD genes using a deep learning model that considers functional characteristics (e.g., gene ontology and expression patterns).

**Results:**

We identified a total of 380 genes achieving statistical significance (5% false discovery rate), including 31 genes affected by de novo CNVs. Of the 380 genes, 52 have not previously been reported as NDD genes, and the data of de novo CNVs contributed to the significance of three genes (*GLTSCR1*, *MARK2*, and *UBR3*). Among the 52 genes, we reasonably excluded 18 genes [a number almost identical to the theoretically expected false positives (i.e., 380 × 0.05 = 19)] given their constraints against deleterious variants and extracted 34 “plausible” candidate genes. Their validity as NDD genes was consistently supported by their similarity in function and gene expression patterns to known NDD genes. Quantifying the overall similarity using deep learning, we identified 11 high-confidence (> 90% true-positive probabilities) candidate genes: *HDAC2*, *SUPT16H*, *HECTD4*, *CHD5*, *XPO1*, *GSK3B*, *NLGN2*, *ADGRB1*, *CTR9*, *BRD3*, and *MARK2*.

**Conclusions:**

We identified dozens of new candidates for NDD genes. Both the methods and the resources developed here will contribute to the further identification of novel NDD-associated genes.

**Supplementary Information:**

The online version contains supplementary material available at 10.1186/s13073-022-01042-w.

## Background

Whole-exome sequencing (WES) enabling comprehensive detection of de novo mutations (DNMs) in protein-coding regions has revealed many novel causative genes of neurodevelopmental disorders (NDDs) [1–4]. However, studies have suggested that many NDD-associated genes still await discovery [1, 2]. Such unidentified genes could be discovered by first developing more sophisticated methods for statistical analysis and second increasing the sample size.

To robustly identify genes responsible for NDDs, the enrichment of DNMs in affected individuals should be statistically evaluated. For this purpose, an approach comparing the observed and expected numbers of DNMs referring to the theoretical DNM rate is often utilized. Specifically, Samocha et al. developed a model of rates of de novo single-nucleotide variants (dnSNVs) considering the trinucleotide context (e.g., a high rate of transitions at CpG sites) and calculated the theoretical per-gene mutation rates of SNVs [5]. Enrichment analyses of dnSNVs using this model of theoretical mutation rates have identified a number of novel disease-causing genes [4]. By contrast, a model of theoretical per-gene mutation rates has not yet been established for copy number variations (CNVs), another class of important disruptive genetic variation. Therefore, previous studies of DNMs aiming at identifying novel causative genes could not integrate information of dnSNVs and de novo CNVs (dnCNVs) in a uniform manner [2].

**Fig. 1:**
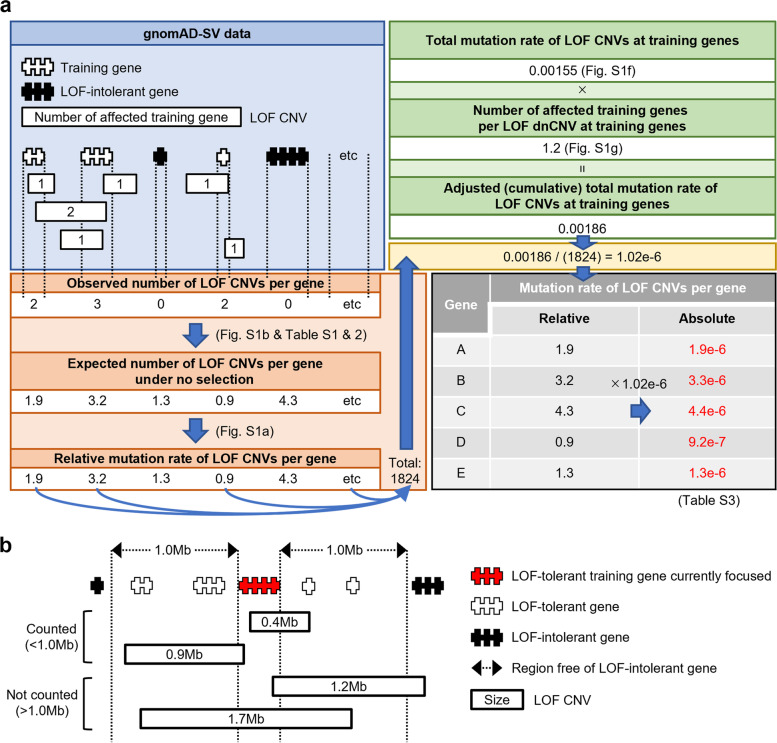
Framework for estimating mutation rates of < 1 Mb LOF CNVs per gene. **a** A conceptional overview showing the method for calculating the mutation rates of < 1 Mb LOF dnCNVs per gene. **b** A scheme depicting the method for selecting training genes. We selected training genes (here, the gene in red) that are LOF-tolerant and flanked by upstream and downstream > 1 Mb regions without any LOF-intolerant genes

To identify new NDD-associated genes, we first addressed the problem in this study by developing a model estimating per-gene rates of dnCNVs considering several factors, such as gene length, number of exons, and information on segmental duplications (SDs) (Fig. 1). Subsequently, we compiled comprehensive lists of dnSNVs and dnCNVs in 41,165 and 3675 individuals with NDDs, respectively, by combining our own new dataset and data from published studies. By using the existing per-gene mutation rates for SNVs, the per-gene mutation rates for CNVs developed in this study, and the comprehensive lists of dnSNVs and dnCNVs from this and previously reported studies, we identified a large number of novel NDD-associated genes achieving exome-wide significance. We also propose a framework to assess the validity of new candidates for disease genes and to prioritize them by integrating various information.

## Methods

### Sample collection

We analyzed 13,851 individuals including 2536 trios in which the child was affected by rare diseases. They were recruited for genetic diagnosis and various studies, such as on autism spectrum disorder (ASD) [6] or epileptic encephalopathy [7] and series of case reports [8, 9], from hospitals in Japan and other countries including Malaysia, Vietnam, Israel, Iran, Turkey, Brazil, Chile, and New Zealand (termed as Yokohama City University [YCU] samples). In a subset of these trios, the child was diagnosed by attending physicians as having an NDD such as intellectual disability (*n* = 1317). Written informed consent was obtained from all of the participants or their guardians. In addition, NDD cases in publicly available data such as a previous Deciphering Developmental Disorders (DDD) study (DDD31k, *n* = 31,058) [1], denovo-db (*n* = 8790) [10], and Simons Simplex Collection (SSC, *n* = 2385) [11] were included in this study (see “Analysis of DDD31k and denovo-db data” below and Additional file 1: Supplementary Methods). The total number is 41,165 (1317 + 31,058 + 8790) for the analysis of SNVs and small indels while 3675 for CNVs (1298 + 2377) because 19 (1317–1298) samples did not pass CNV-based quality checks (QCs). For two samples with a de novo c.662A>G variant in *PIP5K1C*, their phenotypes were collected by attending physicians and described in detail (see “Case reports” in Additional file 1: Supplementary Methods). This study was approved by the institutional review board of Yokohama City University School of Medicine.

### Genetic drift simulation

We performed genetic drift simulation of a gene under a constant population size model by using a custom script (https://github.com/hamanakakohei/AmJHumGenet2020/blob/master/fig.s1.sh.R), as previously described [12–14]. Parameters of the gene were set as follows: selection coefficient: 0; dominance coefficient: 0; constant population size: 100,000; generations: 1,000,000; number of mutation sites (de novo events) in the gene: 100, 320, or 1000; and mutation rate per mutation site in the gene: 1.0e-10, 1.0e-9, 1.0e-8, or 1.0e-7. The settings of the selection coefficient and the dominance coefficient indicate that the variants were under no natural selection. Each simulation generated the number of variant sites observed in 10,000 individuals randomly selected from the last generation, which was about the sample size of Genome Aggregation Database (gnomAD) structural variation (gnomAD-SV) [12, 15]. We set the mutation rate at each mutation site as 1e-7 or less, assuming that CNV mutation rates are likely lower than SNV mutation rates (from 8.0e-10 to 1.2e-7) [16, 17]. This is because the total number of dnCNVs per individual was far lower than that of dnSNVs, even though possible mutation sites of CNVs are far more numerous than those of SNVs [16, 18, 19]. The simulation was repeated > 300 times for each parameter setting. Across the simulations with the same parameter setting, the numbers of variant sites in the gene that were observed in the 10,000 individuals in the last generation were averaged. This average was compared with the total mutation rate in the gene [= (the number of mutation sites in the gene) × (the mutation rate per mutation site in the gene)].

### Regression analyses predicting the number of < 1 Mb loss-of-function (LOF) CNV sites per gene in gnomAD-SV

We performed binomial regression analyses of the number of 50 to 1 Mb LOF (deleting exonic regions) CNVs per gene in gnomAD-SV (https://gnomad.broadinstitute.org/downloads/gnomad_v2_sv.sites.bed.gz) [15] by using a script (CnvModelConstruction_FigS1.R) on our GitHub repository [20]. The lower cut-off of the CNVs was derived from that in gnomAD-SV and a previously proposed definition [21]. For each gene, we used transcripts that were as follows: (1) in GENCODE (gencode.v19.annotation.gff3.gz) and (2) annotated as canonical and protein-coding in snpEff (*n* = 20,034). Among the genes, we selected LOF-tolerant genes flanked by 1 Mb regions free from LOF-intolerant genes at autosomes as training genes (*n* = 2225) (Additional file 1: Fig. 1a and Additional file 2: Table S1). We defined LOF-intolerant and LOF-tolerant genes as genes at the first decile of loss-of-function observed/expected upper bound fraction (LOEUF) (< 0.278), a metric for intolerance to LOF (gnomad.v2.1.1.lof_matrics.by_transcript.txt.bgz), and the other genes (0.278 or more), respectively [19]. For the regression analysis, we used the number of < 1 Mb LOF CNVs per gene in gnomAD-SV as a response variable. As explanatory variables, we used gene length, transcript length, number of exons, distance from the telomere, and number of SD pairs involving or surrounding the gene that are < 1 Mb apart. Locations of SDs were downloaded from the UCSC Genome Browser [22]. We set the distance from the telomere as 25 Mb when it was > 25 Mb because the numbers of < 1 Mb LOF CNV sites at genes in that range appeared to be constant. Despite setting this variable as 25 Mb, the distance from the telomere and the count of < 1 Mb LOF CNV sites showed a negative correlation (*r* = −0.069, *p* = 1.2e-3).

### Correlation between o/e ratios of LOF SNV and LOF CNV sites

We analyzed the correlation between the o/e ratios of LOF SNV sites per gene in gnomAD and < 1 Mb LOF CNV sites per gene in gnomAD-SV by using our script (CnvModelConstruction_FigS1.R) [20]. The o/e ratios of LOF SNV sites per gene in gnomAD were downloaded from the gnomAD portal site (https://gnomad.broadinstitute.org/downloads/gnomad.v2.1.1.lof_metrics.by_transcript.txt.bgz) [19]. The o/e ratios of < 1 Mb LOF CNV sites per gene in gnomAD-SV were calculated based on our model, which predicted the number of < 1 Mb LOF CNV sites per gene in gnomAD-SV (see “Regression analyses predicting the number of < 1 Mb LOF CNV sites per gene in gnomAD-SV”).

### Estimation of the total absolute mutation rate of < 1 Mb LOF CNVs at training genes

We estimated the total mutation rate of < 1 Mb LOF CNVs at the 2225 training genes using the Watterson estimator as the original analysis of gnomAD-SV [15] by using our script (CnvTotalMutationRate_FigS1.R) [20]. Briefly, the Watterson estimator calculates the total mutation rate of CNVs from K (the number of polymorphic sites in a population), Ne (the effective population size), and *n* (the number of alleles). We calculated this estimate in each ethnicity in gnomAD-SV—African/African-American, Latino, East Asian, European, and other populations—and averaged all of the estimates. To experimentally validate the average estimate, we used the total mutation rate of < 1 Mb LOF CNVs at the 2225 training genes in ASD probands or healthy siblings of 519 SSC quads, whose dnCNVs were detected with the gnomAD-SV pipeline and confirmed by quantitative polymerase chain reaction (qPCR) in a previous study [23].

### Mutation rates of SNVs and small indels per gene

We calculated the mutation rates of SNVs and small indels per gene using the trinucleotide-context model (https://github.com/pjshort/dddMAPS) [17], which gives mutation rates in each trinucleotide context, as described in our previous report (script: https://github.com/hamanakakohei/AmJHumGenet2020/blob/master/table.s1.s2.fig.s2a.1.R) [13, 20]. Briefly, the functional category (e.g., missense) of all possible SNVs in each gene was annotated with SnpEff [24]. Then, the per-gene rates of synonymous, missense, and nonsense mutations were determined by summing the rates of all possible variants in one of the three categories in each gene. For the calculation of the mutation rate of frameshift indels, we multiplied the mutation rates of nonsense variants per gene by 1.1, which was the ratio of frameshift to nonsense DNMs identified by exome sequencing in a previous DDD study [3]. Because sequencing depth can affect the number of observed variants, we adjusted the mutation rate at each base according to the median depth per nucleotide, as previously performed [13]: mutation rate × 0.025 × depth, when depth was < 40. The formulas were generated from a curve of the median depth plotted against the ratio of the mutation rate of synonymous variants per nucleotide to the observed number of de novo synonymous variants per nucleotide in our previous study [13]. For the depth adjustment, we randomly selected 100 samples analyzed with respective SureSelect Human All Exon V4, 5, or 6 Kit in YCU and calculated the median depth per nucleotide for the respective kits using samtools. For DDD31k and denovo-db data [1, 10], we could not obtain the median depth per nucleotide and could not adjust the mutation rates per gene with the median depth per nucleotide. Instead, for DDD31k and denovo-db data, to match the expected and observed numbers of DNMs in each set of data, we multiplied per-gene mutation rates by 0.76 and 0.67 for DDD31k and denovo-db data, respectively, which were the o/e ratios of rare (< 0.001% minor allele frequency in 5575 healthy YCU samples and the “non-neuro” subset of gnomAD) synonymous DNMs in each dataset (6028/7938 in DDD31k data and 1502/2246 in denovo-db data).

### Analysis of DDD31k and denovo-db data

We downloaded DNMs detected in the latest DDD31k study [1] (https://www.biorxiv.org/content/10.1101/797787v2) and denovo-db [10] (https://denovo-db.gs.washington.edu/denovo-db.non-ssc-samples.variants.v.1.6.1.vcf.gz and denovo-db.ssc-samples.variants.v.1.6.1.vcf.gz in http://denovo-db.gs.washington.edu/denovo-db/Download.jsp). We excluded “DDD_2017” and “Lelieveld2016” data from denovo-db due to their overlapping of samples with data in the latest DDD31k study. Because denovo-db contained multiple studies using the same samples (i.e., SSC samples in “Iossifov,” “Krumm,” “Turner2016,” “Turner 2017,” and “Werling 2018” and MSSNG samples in “Yuen2016” and “Yuen2017”), we removed duplicated variants in the same sample from these studies, and the total number of samples from these studies turned out to be 2508 in SSC and 1625 in MSSNG, according to the release notes (denovo-db.v.1.6.1.pdf). We excluded studies of target sequencing: “ASD1_2” and “ASD3.” Because denovo-db contained data from multiple studies targeting variable diseases, we only selected studies about “autism,” “congenital_heart_disease,” “developmentalDisorder,” “intellectualDisability,” “epilepsy,” and “sporadic_infantile_spasm_syndrome.” We included the studies about congenital heart diseases because congenital heart diseases and NDDs could coincide, and their genetic causes could overlap [4]. Consequently, we used the following data in denovo-db: SSC data (*n* = 2508), MSSNG data (*n* = 1625), “Hashimoto2015” (*n* = 30), “Homsy2015” (*n* = 1213), “Sifrim2016” (*n* = 859), “Michaelson2012” (*n* = 10), “DeRubeis2014” (*n* = 1445), “Lifton” (*n* = 362), “deLigt2012” (*n* = 100), “epi4k2013” (*n* = 264), “Halvardson2016” (*n* = 39), “Veeramah2013” (*n* = 10), “Rauch2012” (*n* = 51), “Helbig2016” (*n* = 254), “Tavassoli2014” (*n* = 1), “Lee2014” (*n* = 1), “Veeramah2012” (*n* = 1), “Moreno-Ramos2015” (*n* = 4), “Barcia2012” (*n* = 3), and “Dimassi2015” (*n* = 10).

### Enrichment analyses of DNMs per gene

**Fig. 2:**
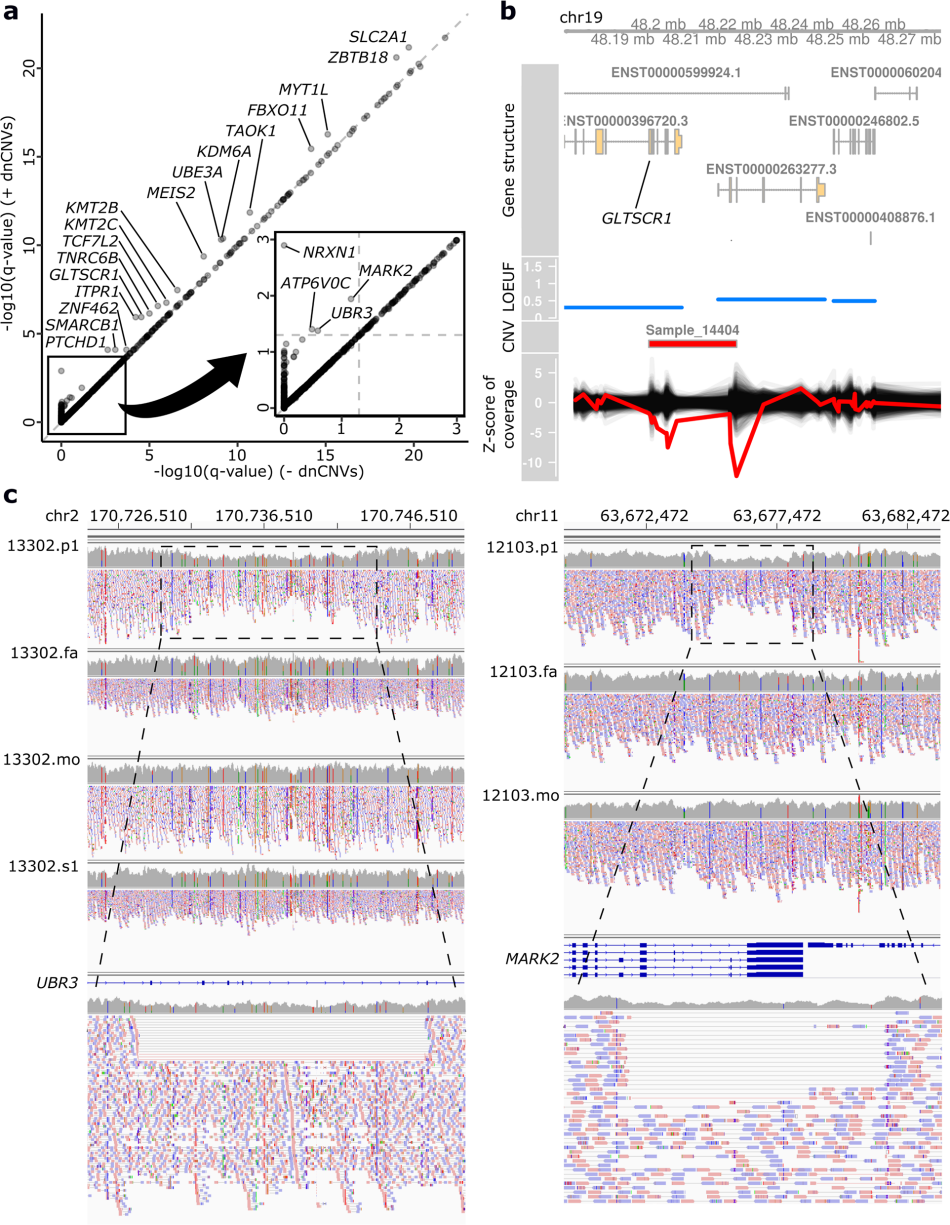
Contribution of dnCNVs to statistical significance of DNM enrichment analyses. **a** A plot of q-values of DNM enrichment analyses for each gene before (*x*-axis) and after (*y*-axis) combining dnCNV data. The gray diagonal line indicates the line of *y* = *x*. The small inset is a magnified image. The dotted lines in the small inset: thresholds for exome-wide statistical significance (*q*-value = 0.05). **b** Visualization of the LOF dnCNVs affecting *GLTSCR1* in a YCU case. From top to bottom, the plots show the exon–intron structures of the canonical transcripts, LOEUF, CNVs called by the exome hidden markov model (XHMM), and *z* scores of depth in the XHMM analysis. LOEUF of each gene is shown as a horizontal line corresponding to its genomic region. In the plot of *z* score for depth, the red line indicates the *z* score of the case with the LOF dnCNV, and the black lines indicate the *z* scores of 500 randomly selected control individuals. **c** IGV images of WGS data of a family with a *UBR3* dnCNV (13302) and a family with a *MARK2* dnCNV (12103). At the top, coverage and paired-end reads of all family members and exon–intron structures of genes are shown. At the bottom, magnified images of coverage and paired-end reads of the affected proband are shown. In the magnified images, discordant read pairs, whose read one and read two surround a dnCNV, are connected with a black line, and split reads, which span a breakpoint, are connected with a red line. p1, the affected proband; fa, the father; mo, the mother; s1, the healthy sibling

The enrichment of DNMs per gene was analyzed by testing the null hypothesis that the observed number of DNMs follows a Poisson distribution whose mean is equal to the expected number of DNMs (script: DnvEnrichment_Fig2FigS4.R) [20]. The expected number of DNMs was calculated as follows: (mutation rate of DNMs per gene in an individual) × (number of analyzed trios). Three patterns of enrichment analyses of DNMs were performed: (1) only LOF analysis, (2) only d-MIS analysis, and (3) LOF + d-MIS analysis. The threshold of the *p*-value for statistical significance was Bonferroni-corrected for the total number of tests: 0.05/(20,034 × 3) (= 8.3E-7).

### Plotting of DNMs

We plotted the locations of de novo SNVs and small indels along with gene structures, read depth per nucleotide in WES in gnomAD, moving average of missense counts per nucleotide in WES in gnomAD, and Pfam domain locations using our script (DnmPlot_FigS7.R) [20]. We downloaded the gene structures from GENCODE (gencode.v19.annotation.gff3.gz) [25], the depth and number of missense variants per nucleotide from the gnomAD portal site (gnomad.exomes.r2.1.coverage.tsv.bgz and gnomad.exomes.r2.1.1.sites.vcf.bgz) [19], and Pfam domains from the Table Browser of the UCSC Genome Browser [22, 26]. To calculate the moving average of the number of missense variants per nucleotide, we annotated the gnomAD variants with SnpEff [24], counted the missense variants at each base, and calculated the moving average of the counts in the surrounding seven bases on each side (15 bases in total).

### Spatiotemporal gene expression analyses

We analyzed whether genes preferentially expressed in each tissue or cell type were enriched in the DNM-enriched genes using the tissue-or cell-specific expression analysis (TSEA or CSEA) tools [27]. The gene expression data for human tissues and human brain subregions were derived from RNA-seq data of the Genotype-Tissue Expression (GTEx) project and the BrainSpan Atlas, respectively [28, 29]. The lists of specifically expressed genes are contained in the specificity index probability (pSI) package of R (http://genetics.wustl.edu/jdlab/psi_package/pSI.data_1.0.tar_.gz/data/human.rda) [30]. The specificity was represented as a pSI score, with a smaller score indicating higher specificity. Whether genes specifically expressed in each tissue or cell type were enriched in the DNM-enriched genes was analyzed using one-tailed Fisher’s exact tests followed by Benjamini–Hochberg (BH) adjustments. We also analyzed whether genes of respective co-expression modules were enriched in the DNM-enriched genes using hypergeometric tests followed by BH adjustments. The modules were previously constructed from microarray data of 1061 samples of various brain regions (frontal, temporal, parietal, occipital, subcortical, and cerebellar regions) at various ages (from 6 weeks post-conception to 30 years of age) by weighted gene co-expression network analyses [31].

### Gene ontology (GO) analyses

We analyzed which GO terms were enriched in the DNM-enriched genes using ToppGene [32] by using our script (GeneOntology_Fig4.py) [20]. We analyzed three different GO terms: GO biological process (BP), GO molecular function (MF), and GO cellular component (CC). We analyzed only GO terms with 20 to 500 genes because GO terms with > 500 genes are less specific and give little insight into the genes’ functions. Clustering of the enriched GO terms according to the similarity between terms was performed using the EnrichmentMap plugin of Cytoscape [33, 34]. A gene pair was connected by an edge when the Jaccard and overlap combined coefficient was greater than 0.375. The functions of clusters of ten or more GO terms were manually annotated. Edge width represents the overlap coefficient, and node size represents the number of genes belonging to the node. After clustering GO terms, we inspected each cluster and manually annotated them with what the cluster represents.

### Protein-protein interaction (PPI) network analysis

We used Search Tool for the Retrieval of Interacting Genes/Proteins (STRING), a comprehensive database of PPIs. STRING provides information about clusters of interacting human proteins (hereafter, STRING clusters), annotated with several databases such as Reactome and UniProt [35]. To analyze which STRING cluster’s components are enriched in the proteins encoded by the DNM-enriched genes, we downloaded cluster descriptions (clusters.info.v11.0.txt.gz) and protein members of all clusters (clusters.proteins.v11.0.txt.gz) from the STRING web server [35], and then calculated *p*-values for each cluster using hypergeometric tests and corrected them for multiple testing with the BH method by using our script (String.py) [20].

### Preprocessing of predictors for neural network (NN) model construction

We used seven predictors for NN model: the probability of being loss-of-function intolerant (pLI) [16, 19]; LOEUF, a conservative estimate of the observed/expected ratio [19]; missense *z*-score of the observed missense counts compared to expected [16, 19]; and the results of TSEA, brain subregion/stage-specific expression analysis, co-expression module analysis, and STRING analysis. We preprocessed these as follows: For the TSEA results, we set genes with pSI < 0.05 in the brain as one and the other genes as zero. For brain subregion/stage-specific expression analysis results, we used the number of brain subregions/stages with pSI score < 0.05 among the brain subregions/stages enriched in the 328 known genes. For co-expression module analysis results, we regarded this variable as categorical and set modules except for M1, M4, M7, and M13 as “Others.” For GO analysis results, we used the number of GO terms enriched in the 328 known genes. For STRING analysis results, we set genes that are a member of STRING clusters enriched in the 328 known genes as one and the other genes as zero.

### NN model construction

We designed the model architecture, trained the model, and evaluated the model using the Keras application programming interface of the TensorFlow2 package in Python3 by using our script (Fig.6bcd_S9_TableS15.py) [20, 36]. We trained an NN comprising one input layer (8 neurons), two hidden dense layers with rectified linear unit functions (128 neurons), and one output layer with a sigmoid function (1 neuron). The last sigmoid function outputs a score of 0-1. We used Adam as the model optimization algorithm and binary cross entropy as the loss function. For training, the parameters of the model were updated in each of the five genes. The model was trained for the whole training gene set five times.

### AUC comparison

We determined the area under the receiver operating characteristic curve (AUC) of our NN model, eight predictors, and three metrics for PC3 and NC3 by using our script (Fig.6bcd_S9_TableS15.py) [20]. Empirical *p*-values of the difference in AUC between our NN model and others were based on a distribution of the AUC of 500 NN models. The *p*-values were further adjusted by the BH method. The three metrics were residual-variance intolerance score (RVIS) (http://genic-intolerance.org/data/RVIS_Unpublished_ExACv2_March2017.txt) [37], which indicates intolerance to functional variations, gene damage index (GDI) (https://lab.rockefeller.edu/casanova/GDI/GDI_full.txt) [38], which indicates the load of functional variations in the general population, and Human Gene Connectome (HGC) [39, 40], which measures the biological distance between two genes. For the HGC score, we manually give NDD genes in PC1 (*n*=243) and PC2 (*n*=33) as input for “Core Genes” and genes in PC3 (*n*=246) and NC3 (*n*=1000) for “Genes of Interest” in HGC server (https://hgc.rockefeller.edu/) and obtained the distance of a target gene to each of the 276 known NDD genes and regarded the minimum distance among the 276 scores as the HGC score of the target gene.

### Application of the NN model to the 34 plausible candidate genes

The NN model cannot handle genes for which some of the data are missing. For 6 of the 34 plausible candidate genes, brain subregion/stage-specific expression or co-expression module analysis results were missing. Therefore, we converted the missing data in the brain subregion/stage-specific expression analysis results to the median (i.e., 1) of the 34 genes, while we converted the missing data in the co-expression module analysis results to “Others” (see “Preprocessing of predictors for NN model construction”).

### Calculation of likelihood ratios

To calculate the likelihood ratios of the NN model scores, we approximated the score distributions of PC3 and NC3 using Kernel density estimation implemented in scikit-learn library [41], which was the same algorithm as the violin plots in Fig. 6a, by using our script (Fig.6bcd_S9_TableS15.py) [20]. From the approximate distributions, we obtained probability densities of model scores in NC3 and PC3. By dividing the probability densities in PC3 with those in NC3, we calculated the likelihood ratios of NN model scores.

## Results

### Construction of models predicting the relative per-gene mutation rates of LOF CNVs

We first aimed to construct a model calculating the mutation rates of LOF CNVs per gene (only focusing on deletions affecting one or more exons, but not including other types of LOF SVs such as intragenic duplications and mobile element insertions) [15] (Fig. 1). Previous studies demonstrated that the mutation rates of synonymous SNVs per gene correlate with the number of synonymous SNV sites per gene in the general populations of the Exome Aggregation Consortium (ExAC) and gnomAD [16, 19], despite various local mutation rates at each base. From this finding, we hypothesized that the mutation rates of variants at selectively neutral loci correlate with the counts of variant sites in a population, and thus, the de novo mutation rates of LOF CNVs per neutral gene can be estimated from the number of LOF CNV sites per gene in the gnomAD-SV dataset. To prove this, we performed simulations of mutational events and genetic drift through generations under various parameter settings [12, 13]. The simulations showed that mutation rates per gene and numbers of variant sites per gene observed in gnomAD-SV closely correlate when the variants are not under natural selection (Additional file 1: Fig. S1a).

The above result indicates that relative mutation rates of LOF CNVs per gene can be calculated from the number of LOF CNV sites per gene in gnomAD-SV, when the LOF CNVs are not under selection. We subsequently selected genes in such regions where LOF CNVs are unlikely to be under selection using the following criteria: (1) genes that are tolerant of LOF variants, as indicated by the LOEUF score [19] > 0.278 (genes not belonging to the most constrained decile) and (2) genes flanked by 1 Mb upstream and downstream regions without any LOF-intolerant genes (LOEUF < 0.278) (Fig. 1b). We then used the data of LOF CNVs that are smaller than 1 Mb in gnomAD-SV (“< 1 Mb LOF CNVs” in the following) to count the numbers of LOF CNV sites in these genes (Fig. 1b).

Using these genes as the training set, we next constructed a model predicting the number of < 1 Mb LOF CNV sites per gene in gnomAD-SV, which corresponds to relative de novo mutation rates of < 1 Mb LOF CNVs per gene as simulated above. For this purpose, we tested the following six variables possibly correlated to the number of CNV sites per gene: gene length, number of exons, transcript length, number of SD pairs involving or surrounding a gene, distance from the centromere, and distance from the telomere (Additional file 2: Table S1). We selected the latter three because SD pairs could generate CNVs via non-allelic homologous recombination and genomic regions near the centromere and the telomere had more CNVs [15, 42]. As expected, gene length, number of exons, and transcript length positively correlated with the number of < 1 Mb LOF CNV sites in the training genes (*n* = 2225) in gnomAD-SV (Additional file 1: Fig. S1b). The number of SD pairs involving or surrounding a gene also showed a significant positive correlation, but with a non-linear pattern (Additional file 1: Fig. S1b). The distance from the centromere showed a weak correlation with a fluctuation of the moving average. The distance from the telomere showed a negative correlation in the regions near the telomere (0–25 Mb, the left side of the black dotted vertical line in the rightmost panels of Additional file 1: Fig. S1b), whereas the correlation between per-gene < 1 Mb LOF CNV sites and the distance from the telomere was unclear in the distal regions (> 25 Mb, the right side of the black dotted vertical line in the rightmost panels of Additional file 1: Fig. S1b). From these results, we decided to use gene length, number of exons, transcript length, number of SD pairs, and distance from the telomere for the regions near the telomere (see “Methods”) as the explanatory variables. Using all possible combinations of these five explanatory variables (2^5^–1 = 31), we performed binomial regression analyses and constructed 31 models predicting the number of < 1 Mb LOF CNV sites per gene based on the training genes (Additional file 2: Table S2).

### Selection and validation of the best model

Among the 31 models constructed above, the 4th model considering gene length, number of exons, number of SD pairs involving or surrounding a gene, and distance from the telomere had the lowest AIC. We selected this model as the best one and used it for further analyses (Additional file 2: Table S2).

On the basis of this model, we predicted the number of CNV sites per gene in all genes, including those not in the training set. The observed number of CNV sites in gnomAD-SV and the expected number of those from the 4th model are highly significantly correlated in the training genes (Pearson’s correlation coefficient: 0.38, *p*-value = 3.0e-79; Additional file 1: Fig. S1c). A highly significant correlation with a smaller correlation coefficient was also observed among the other (non-training) genes (Pearson’s correlation coefficient: 0.24, *p*-value = 1.4e-229; Additional file 1: Fig. S1c). These results suggested that the model works for all genes. When comparing the training genes with the non-training genes, the expected numbers were less than the observed numbers only in the non-training genes. This is reasonable because the non-training genes include LOF-intolerant ones where LOF CNVs should be under natural selection. We also performed validation of our model using the ratios of observed numbers of LOF CNV sites in gnomAD-SV to the expected numbers based on our model (o/e ratios). The o/e ratio of LOF CNVs for a gene is expected to be lower when the natural selection against LOF CNVs hitting the gene is stronger. This should also be true for the o/e ratio of LOF SNVs because LOF CNVs and SNVs are considered to damage gene function equally. Therefore, we investigated the correlation between the o/e ratios of LOF SNV sites in gnomAD and LOF CNV sites in gnomAD-SV. We found that these two ratios are highly significantly correlated (*r* = 0.20, *p*-value = 5.9E-168; Additional file 1: Fig. S1d). In addition, the o/e ratio of LOF CNV sites negatively correlated with a gene-level score of intolerance to deletions (gs://gcp-public-data—gnomad/legacy/exacv1_downloads/release0.3.1/cnv), which was computed based on ExAC WES data (Pearson’s *r* = -0.24, p = 1.2e-120; Additional file 1: Fig. S1e) [43]. These results collectively demonstrate that the model reasonably predicts theoretical counts of LOF CNV sites per gene on the assumption that the LOF CNVs are under no selection.

In these validations, the 4th model considering multiple explanatory factors showed lower *p*-values than simpler models based on gene length (the 27th model) or transcript length (the 29th model) (Additional file 2: Table S2). Therefore, the 4th model, which demonstrated the best performance in predicting per-gene LOF CNV rates in the training genes, is thought to also be superior to the simpler models in predicting the non-training genes. On the basis of this 4th model, we obtained the relative per-gene mutation rates of LOF CNVs in all genes.

### Conversion of the relative per-gene mutation rates of LOF CNVs to the absolute mutation rates for DNM enrichment analysis

Using the best model described above (the 4th model), we can estimate the number of < 1 Mb LOF CNV sites per gene in gnomAD-SV, which should correspond to the relative per-gene dnCNV rates (Additional file 1: Fig. S1a). Meanwhile, we need to convert the relative per-gene rates into the absolute rates to determine the theoretically expected numbers of dnCNVs in each gene in a patient cohort. To this end, we estimated the total absolute mutation rate of < 1 Mb LOF CNVs in the training genes using the Watterson estimator, as previously described (Fig. 1b) [15]. The Watterson estimator, which considers the number of variant sites and the population size in gnomAD-SV, estimated the total absolute mutation rate of < 1 Mb LOF CNVs in the training genes to be 0.00155. This estimate is comparable to the mutation rate in children in the SSC dataset (519 quads), in which CNVs were detected in the same way as for gnomAD-SV and experimentally validated (Additional file 1: Fig. S1f) [15, 23]. In this analysis, we included the probands because we assumed that even the affected children likely have a comparable number of dnCNVs at neutral genes. We further performed an adjustment of the total absolute mutation rate considering that one CNV could affect multiple genes. On average, < 1 Mb LOF CNV sites in gnomAD-SV in the training genes affect approximately 1.2 training genes (Additional file 1: Fig. S1f). On the basis of this number, we calculated the adjusted (cumulative) total mutation rate of < 1 Mb LOF CNVs in the training genes as 0.00186 (0.0155 × 1.2) (Fig. 1a).

From these results, we obtained the following absolute mutation rates of < 1 Mb LOF CNVs per gene for 20,034 genes (protein-coding and canonical transcripts in GENCODE and snpEff) (see “Methods”) (Additional file 2: Table S3) (Fig. 1a): (the total absolute mutation rate in the training genes, 0.00155) × (the number of the training genes affected by dnCNVs in the training genes, 1.2)/(the total relative mutation rate in the training genes, 1824) × (the relative mutation rate of a gene of interest). Because the abovementioned validation of the estimated total mutation rate was performed by using a subset of genes (*n* = 2225) (Additional file 1: Fig. S1f), we repeated the analysis using all genes (*n* = 20,034) whose absolute mutation rates were finally obtained here. By summing up all mutation rates per gene, corresponding to the theoretical per-individual number of genes affected by LOF dnCNVs, and comparing this summed value to the total number of genes affected by experimentally validated LOF dnCNVs in 519 quads in SSC described above, we observed that the theoretically expected number is consistent with the observed number, especially in healthy siblings (Additional file 1: Fig. S1h). This indicates that our estimate of the total mutation rate is reasonable. Thus, we could obtain per-gene mutation rates of all genes that can be used in the DNM enrichment analyses as shown below.

### Gene-based enrichment analyses of dnSNVs and dnCNVs in NDDs

Subsequently, we compiled comprehensive lists of dnSNVs and dnCNVs in NDD cases to maximize the chance of gene discovery. We collected the data of DNMs from the following four datasets: our YCU data for dnSNVs and dnCNVs; the study of ~31,000 developmental disorder cases combining healthcare and research data by the DDD project and others (DDD31k) for dnSNVs [1]; denovo-db, a collection of DNMs assembled from the literature, for dnSNVs [10]; and the SSC dataset for dnCNVs (Additional file 1: Fig. S2) [11]. We combined these data while being aware of the overlaps across the datasets. For example, data of dnSNVs in the SSC dataset were not used as these are included in the denovo-db dataset. Variant- and sample-level QCs of the dnSNVs and dnCNVs in our YCU cohort and dnCNVs in the SSC dataset were performed as described in Additional file 1: Supplementary Methods and Fig. S2 and S3. After the compilation, we included the data of dnSNVs in 1317 cases in YCU and dnCNVs in 1298 cases in YCU, dnSNVs in 31,058 cases in DDD31k, dnSNVs in 8790 cases in denovo-db, and dnCNVs in 2377 cases in SSC quads in the downstream analyses (Additional file 2: Table S4, S5, and S6).

By using the above-described framework for the analysis of dnSNVs and dnCNVs in a uniform manner, and the compiled comprehensive lists of dnSNVs and dnCNVs in NDD cases, we performed enrichment analyses of dnSNVs and dnCNVs. To combine the datasets from different cohorts, we matched the expected and observed numbers of synonymous dnSNVs (see Additional file 1: Fig. S4 and Supplementary Methods). To calculate the total expected count of dnSNVs and dnCNVs in a gene, we added up each expected count because the sum of two Poisson random variables, each with the mean λ_1_ or λ_2_, forms a Poisson random variable whose mean is λ_1_ + λ_2_. We performed the following three patterns of enrichment analysis of DNMs for each gene (*n* = 20,034): (1) only LOF (nonsense, frameshift, splice acceptor site, splice donor site, and CNV) analysis; (2) only d-MIS [damaging missense: missense variants with > 2 Missense badness, PolyPhen-2, and Constraint (MPC [44]) score] analysis; and (3) LOF + d-MIS analysis using the data of both LOF and d-MIS DNMs.

For multiple testing corrections, we performed two-step adjustments. We first applied a gene-level adjustment based on the number of tests for each gene, that is, three for genes with MPC annotation and one for genes with no such annotation, using the Bonferroni method. After that, we selected the smallest Bonferroni-adjusted *p*-value for each gene. Next, we performed an exome-wide adjustment based on the number of tested genes using the BH method to obtain a q-value for each gene. After corrections, we identified a total of 381 genes significantly enriched for DNMs (q-value < 0.05) (Additional file 2: Table S7). Of these 381 genes, we identified dnCNVs in 32 genes (Additional file 2: Table S8), and these dnCNVs contributed to lower q-values (Fig. 2a): *MECP2*, *STXBP1*, *SCN2A*, *EHMT1*, *WAC*, *FOXG1*, *ZBTB18*, *HNRNPU*, *BCL11A*, *SMC1A*, *SLC2A1*, *SMARCB1*, *MYT1L*, *FBXO11*, *TAOK1*, *KDM6A*, *UBE3A*, *KMT2B*, *ITPR1*, *ATP6V0C*, *NRXN1*, *ARID1B*, *CHD2*, *CSNK2A1*, *MEIS2*, *KMT2C*, *TCF7L2*, *TNRC6B*, *ZNF462*, *GLTSCR1*, *MARK2*, and *UBR3*. We confirmed these CNVs in YCU samples by qPCR (Fig. 2b and Additional file 1: Fig. S5) and those in SSC samples by manual inspection with Integrative Genomics Viewer (IGV) (Figs. 2c and S6). We noted that both *ZBTB18* and *HNRNPU* were affected by one dnCNV, and the dnCNVs at *STXBP1*, *EHMT1*, *BCL11A*, *KDM6A*, and *ATP6V0C* also affected other LOF-constrained (> 0.9 pLI and < 0.35 LOEUF) genes such as *RALGPS1* and *ENG*, *ZMYND19*, *PAPOLG* and *REL*, *EFHC2*, and *PDPK1*, respectively (Additional file 1: Fig. S6b, d, g, h, o, and s). According to previous literature, the pathogenicity of *STXBP1*, *EHMT1*, *ZBTB18*, *HNRNPU*, *BCL11A*, and *KDM6A* deletions have been established [45–51]. Meanwhile, the deletion of *PDPK1*, but not *ATP6V0C*, may be truly pathogenic. Therefore, we conservatively excluded the dnCNV count at *ATP6V0C*. After that, the enrichment of DNMs at *ATP6V0C* was no longer statistically significant.

### Identification of plausible candidates for novel NDD genes

Among the 380 genes, in which *ATP6V0C* is not included, we analyzed whether each gene was previously reported as (1) an autosomal dominant or X-linked candidate NDD gene in the DDG2P (Development Disorder Genotype Phenotype Database) [52], (2) an NDD-related haploinsufficient gene in ClinGen Dosage Sensitivity Map (https://ftp.clinicalgenome.org/ClinGen_gene_curation_list_GRCh38.tsv) [53], (3) statistically significant in three previous large studies of DNMs [1, 2, 54], or (4) a gene causative of NDDs in PubMed, excluding case reports (Additional file 2: Table S7). We found that 328 genes fell into at least one of the above categories. Therefore, we defined them as known NDD genes. Meanwhile, no such reports on the remaining 52 genes had been published, indicating that they can be new candidate genes for NDDs (Additional file 1: Fig. S7). Of these 52 candidates, dnCNVs contributed to the significance of *GLTSCR1*, *MARK2*, and *UBR3* (Fig. 2b, c).

Among the 52 candidates, 43 and 26 genes are enriched for LOF and d-MIS DNMs, respectively, in NDDs (17 genes are enriched for both). By evaluating the 43 genes with LOF enrichment in light of their constraints against LOF variants in the general population using two established metrics (the pLI and LOEUF scores) [16, 19], we found that these genes are significantly LOF-constrained compared with the others (Wilcoxon rank sum test *p*-values = 1.6e-8 and 6.8e-5) (Additional file 1: Fig. S8a). This further supports the candidacy of these genes as novel NDD genes and suggests that haploinsufficiency of these genes is the relevant pathomechanism (Additional file 2: Table S9). In particular, genes with strong constraints against LOF variants can be good candidates. We found that there are 23 such genes with pLI > 0.9 and LOEUF < 0.35 (Additional file 2: Table S9). We also noted that *ELAVL3* has very low observed/expected ratios of LOF variants (0.15), while the LOEUF (0.46) and pLI scores (0.77) of this gene were modest, probably due to its small gene size. Similarly, we evaluated the 26 genes enriched for d-MIS variants in NDDs using the missense z-score in gnomAD, an indicator of a constraint against missense variants in the general population. We found that there is an overall bias toward a constraint against missense variants among the 26 genes with d-MIS DNM enrichment (Wilcoxon rank sum test *p*-value = 2.9e-14) (Additional file 1: Fig. S8b). We found that 20 of the 26 genes were highly depleted of missense variants (> 2.5 missense z-score) and were thus considered as good candidate genes (Additional file 2: Table S9). Notably, 7 of these 26 candidates harbor recurrent (affecting the same amino acids) d-MIS DNMs (Additional file 2: Tables S8 and S9), that is, *PSMC3*, *PIP5K1C*, *KIAA0100*, *SEPT2*, *KBTBD7*, *REST*, and *MAST3*. This observation strongly indicates the pathogenicity of these specific variants as well as the association of these genes with NDDs, considering the very low probability of observing multiple DNMs at the same amino acids. Furthermore, we identified another individual, recruited after performing the above enrichment analyses, with the c.662A>G DNM at *PIP5K1C* and found that the individual showed phenotypes such as delayed language acquisition and facial dysmorphism, which are consistent with those of the YCU case of the same variant, included in the enrichment analyses (case report in Additional file 1: Supplementary Results).

On the basis of these observations, we considered the genes meeting either of the following criteria as plausible candidate genes for NDDs: (1) genes enriched for LOF DNMs in NDDs and constrained for LOF variants in the general population or (2) genes enriched for d-MIS DNMs in NDDs and constrained for missense variants in the general population and/or harboring recurrent d-MIS DNMs (*n* = 34, Additional file 2: Table S9). This number of plausible candidate genes, 34, is quite consistent with the expected number of true-positive genes among the 52 candidates for novel NDD genes, which we estimated as 380–328–19 = 33, based on the fact that 328 known NDD genes are highly likely to be true positives and that there should be 19 false positives among the 380 genes, according to the false discovery rate (FDR) used in our analysis (i.e., 380 × 0.05 = 19). We subsequently analyzed the properties of these 34 plausible candidate genes.

### Evaluation of plausible candidate genes by comparison with known NDD genes

To further evaluate the validity of these 34 plausible candidates as novel NDD genes, we performed a series of bioinformatic analyses in which we characterized the properties of the 328 known NDD genes and then tested whether the same or similar characteristics were observed in the 34 plausible candidates.

**Fig. 3:**
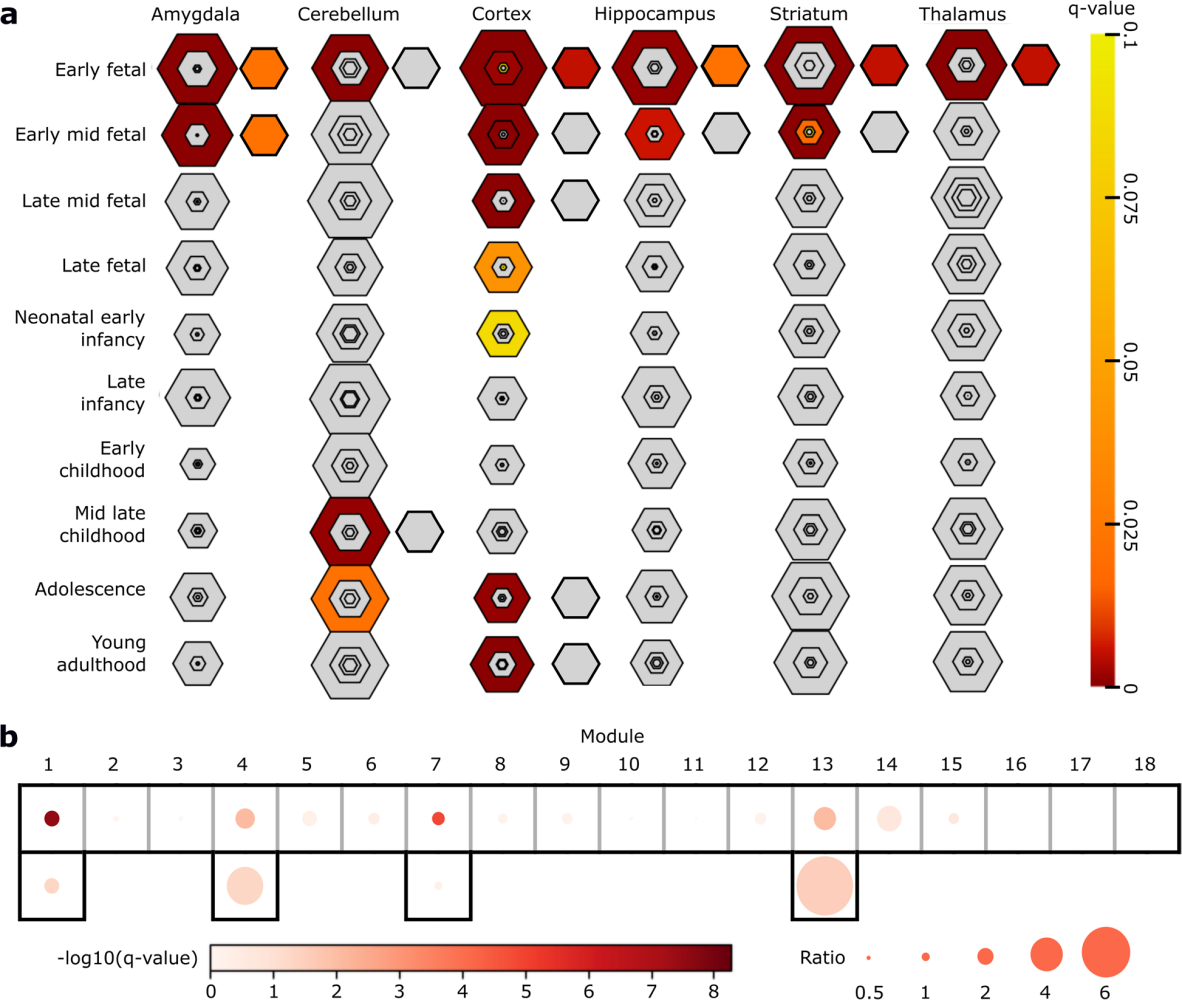
Spatiotemporal expression patterns of the 328 known and 34 plausible candidate genes. **a** Enrichment analyses of genes specifically expressed in each brain region at each developmental stage in the 328 known (the six columns of large hexagons) and 34 plausible new genes (columns of small hexagons on the right of the columns of large hexagons). Sizes of the hexagons for the 328 genes correlate with their gene set sizes. The red colors correspond to q-values of Fisher’s exact tests adjusted by the BH method. The regions of the hexagons for the 328 genes closer to the center of each hexagon correspond to genes with smaller pSI scores, namely, increasing specificity (< 0.05, < 0.01, < 0.001, and < 0.0001, respectively), while the hexagons for the 34 genes correspond to genes with pSI scores < 0.05. **b** Enrichment analyses of genes of each co-expression module in the 328 known (the upper row) and 34 plausible candidate genes (the lower row). The circle colors correspond to q-values of hypergeometric tests adjusted by the BH method. The circle sizes indicate the ratio of each module proportion in the 328 or 34 genes relative to that in all genes

First, we performed an analysis informed by various resources of gene expression patterns. When we tested in which tissues the 328 known genes were preferentially expressed using the data of various human tissues from the GTEx study [28], we found that genes preferentially expressed in the brain (pSI score [27] < 0.05, see “Methods”) are significantly enriched among the 328 genes (*q*-value < 2.0e-4). By contrast, there was no such enrichment in the other 24 tissues (Additional file 2: Table S10). In the 34 genes, we observed a similar pattern: a trend toward the enrichment of brain-specific genes (*p* = 0.07, pSI < 0.01) and no trend toward the enrichment of genes specific to other tissues (Additional file 2: Table S10). To obtain a higher spatiotemporal resolution within the brain, we then used the expression patterns in various brain regions and developmental periods in the BrainSpan atlas [29]. Among the 60 spatiotemporal coordinates (6 brain regions × 10 developmental periods), we found that genes preferentially expressed in 14 coordinates, mainly consisting of broad regions in the fetal period and the postnatal cortex and cerebellum, were enriched in the 328 genes (pSI < 0.05, *q*-value < 0.01) (Additional file 2: Table S11 and Fig. 3a). We then analyzed whether the genes preferentially expressed in these 14 coordinates were also enriched in the 34 plausible candidate genes. The analysis showed significant enrichment in the six coordinates in the fetal period (*q*-value < 0.05), an observation unlikely to have occurred by chance. We also analyzed co-expression modules observed in various brain regions and developmental periods [31]. We found that genes assigned to modules (M)1, 4, 7, and 13 were enriched in the 328 genes (*q*-value < 0.05) (Fig. 3b and Additional file 2: Table S12). Regarding the general characteristics of these modules, M1 is enriched for genes expressed specifically in the fetal period and associated with chromatin organization, M4 is enriched for genes expressed specifically in the fetal and perinatal periods and associated with neuronal differentiation, M7 is enriched for genes expressed across development and associated with RNA processing and splicing, and M13 is enriched for genes expressed preferentially in the cortex and cerebellum from childhood and associated with neuronal excitability [31]. Therefore, it is reasonable to assume that these four modules are associated with the pathomechanisms of NDDs. Among the genes in these four modules, M13 genes were significantly enriched in the 34 plausible candidate genes (*q*-value < 0.05), and M1 and M4 genes showed nominal significance (*q*-values = 0.053) (Fig. 3b). Taken together and considering the smaller number of plausible candidate genes when compared with known genes, these results indicate that the 328 known and the 34 plausible candidate genes exhibit similar expression characteristics.

**Fig. 4:**
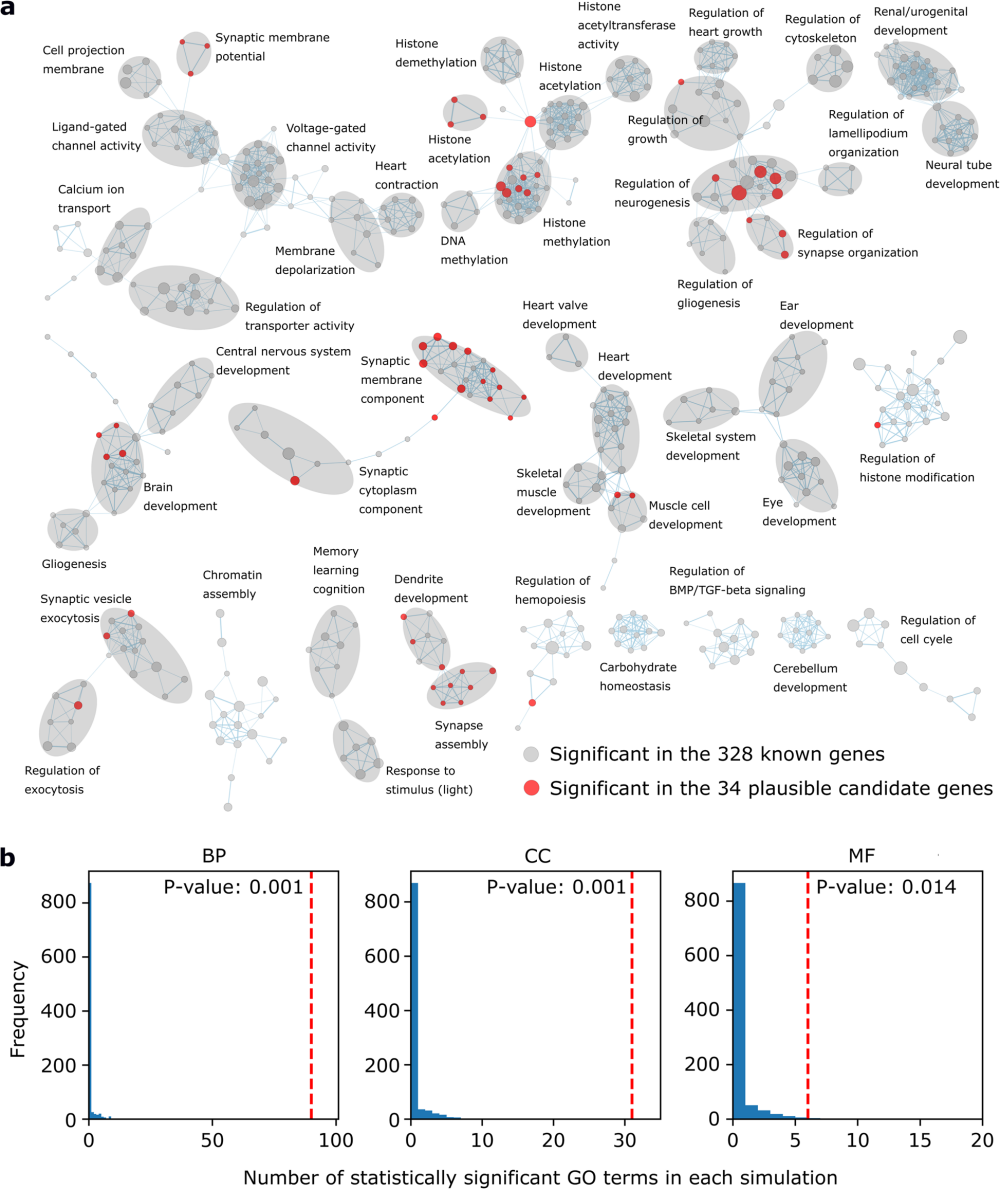
GO terms enriched in the 328 known and 34 plausible candidate genes. **a** Clusters of GO terms enriched (*q*-value < 0.01) in the 328 known and 34 plausible candidate genes. Only clusters of ten or more nodes are shown. Each node represents a GO term. Nodes are connected by an edge when the Jaccard and overlap combined coefficient for their gene members is > 0.5. Node size represents the number of gene members. Nodes are colored red when the nodes are statistically significant in the 34 plausible candidate genes. Gray ovals represent manually annotated GO groups. **b** Histograms of numbers of GO terms enriched (*q*-value < 0.01) in 34 randomly selected genes. This simulation was repeated 1000 times. In each simulation, only the 1086 terms enriched in the 328 known genes (Additional file 2: Table S13) were analyzed. Red bars indicate the number of GO terms enriched in the 34 plausible candidate genes. Empirical p-values of the enrichment in the 34 genes are the proportion of simulations with a number of GO terms equal to or more than that of the red bars. BP, GO biological process terms; CC, GO cellular component terms; MF, GO molecular function terms

Second, we investigated the similarity between the 328 known and 34 plausible candidate genes in terms of their biological properties by performing GO enrichment analyses using ToppFun [32]. Among the 328 known genes, we observed significant (*q*-value < 0.01) enrichment of 1086 terms, comprising 843 BP, 130 CC, and 113 MF terms (Additional file 2: Table S13) [32]. When we visualized this result by clustering GO terms containing similar genes using Cytoscape (Fig. 4a) [33], we observed multiple clusters of terms associated with neuron or brain development, such as “regulation of neurogenesis” and “central nervous system development,” as well as clusters of terms associated with processes that are related to NDD pathogenesis, such as “histone methylation” and “synapse assembly.” We subsequently investigated whether the 1086 NDD-associated terms were enriched in the 34 plausible candidate genes. Of the 1086 terms, 90 BP, 31 CC, and 6 MF terms were enriched at *q*-value < 0.1 (Fig. 4, red nodes). By statistically evaluating these observed numbers of BP, CC, and MF terms, we found that all of them are unlikely to be observed by chance (empirical *p*-values = 0.001, 0.001, and 0.014, respectively; calculated by 1000× random sampling of 34 genes) (Fig. 4b). Therefore, it was considered that the biological properties of the 34 plausible candidate genes are similar to those of the 328 known genes.

**Fig. 5:**
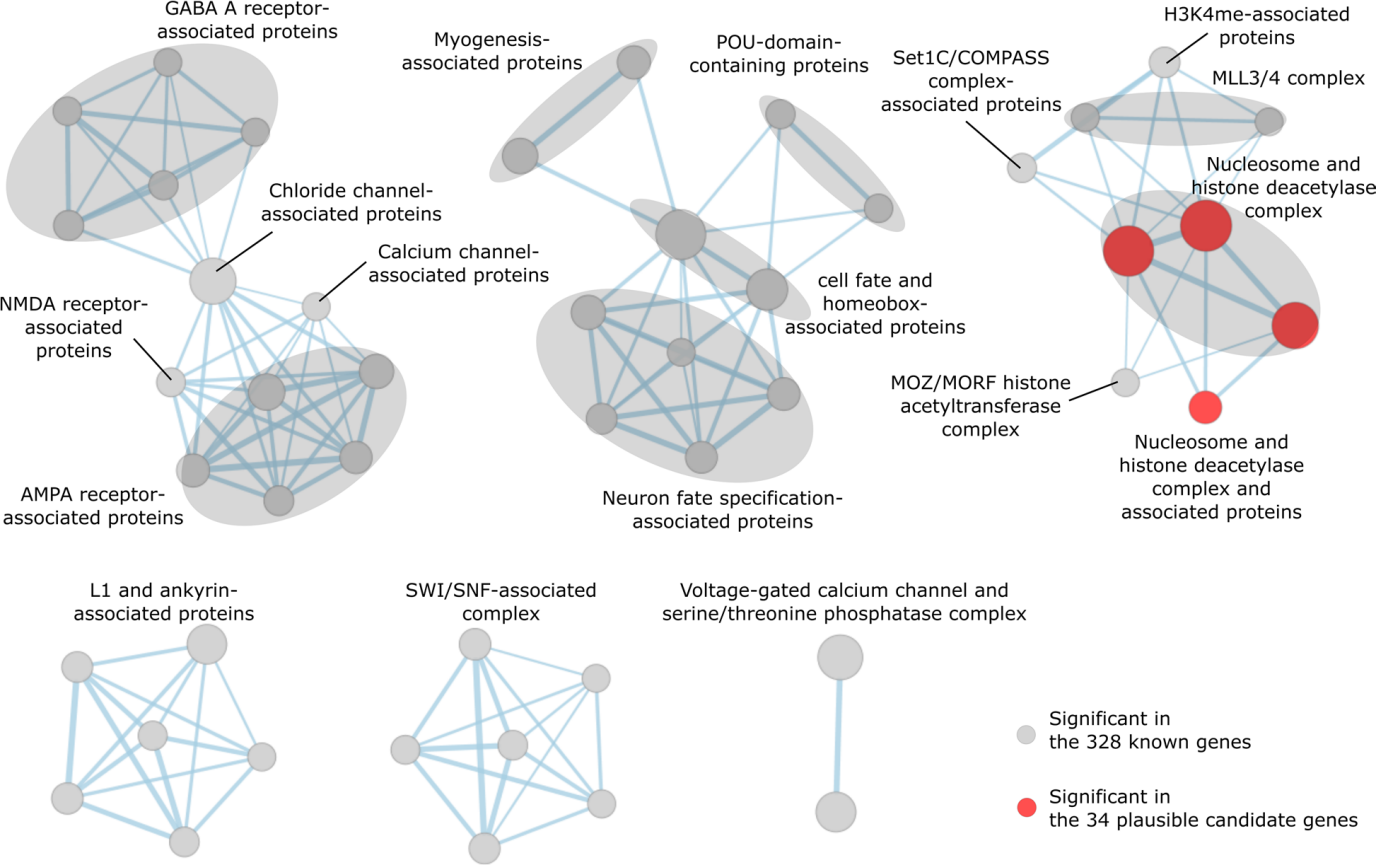
STRING clusters enriched in the 328 known and 34 plausible candidate genes. STRING clusters whose members are enriched (*q*-value < 0.01) in the proteins encoded by the 328 known and 34 candidate genes. Nodes are clustered according to the similarity of their members. Nodes are connected by an edge when the Jaccard and overlap combined coefficient for their members is > 0.375. Gray nodes: STRING clusters significantly enriched in the 328 known genes; red nodes: STRING clusters significantly enriched in the 34 candidate genes. Gray ovals: groups of nodes with similar annotations

Third, we performed a protein-level analysis because proteins encoded by NDD-associated genes are highly interconnected within PPI networks [2, 55]. Clusters of interacting human proteins are provided in STRING, a comprehensive database of PPIs [35]. We analyzed whether the component proteins of each STRING cluster significantly overlap with the proteins encoded by the 328 known genes. We identified 54 STRING clusters with significantly more overlaps (hypergeometric test *q*-value < 0.01) (Additional file 2: Table S14 and Fig. 5). Of these 54 STRING clusters, we found that component proteins in four clusters (cluster ID: 11115, 11116, 11117, and 11339) overlapped with the 34 plausible candidates at *q*-value < 0.1 (red nodes in Fig. 5 and Additional file 2: Table S14). This observed number of overlaps (4 out of 54) was, again, statistically significant when compared with the expectation from 1000× random sampling of 34 genes (empirical *p*-value = 0.008). Thus, protein-level evidence also supports the validity of the 34 plausible candidate genes as NDD genes.

### Prioritization of plausible candidate genes using deep learning

**Fig. 6:**
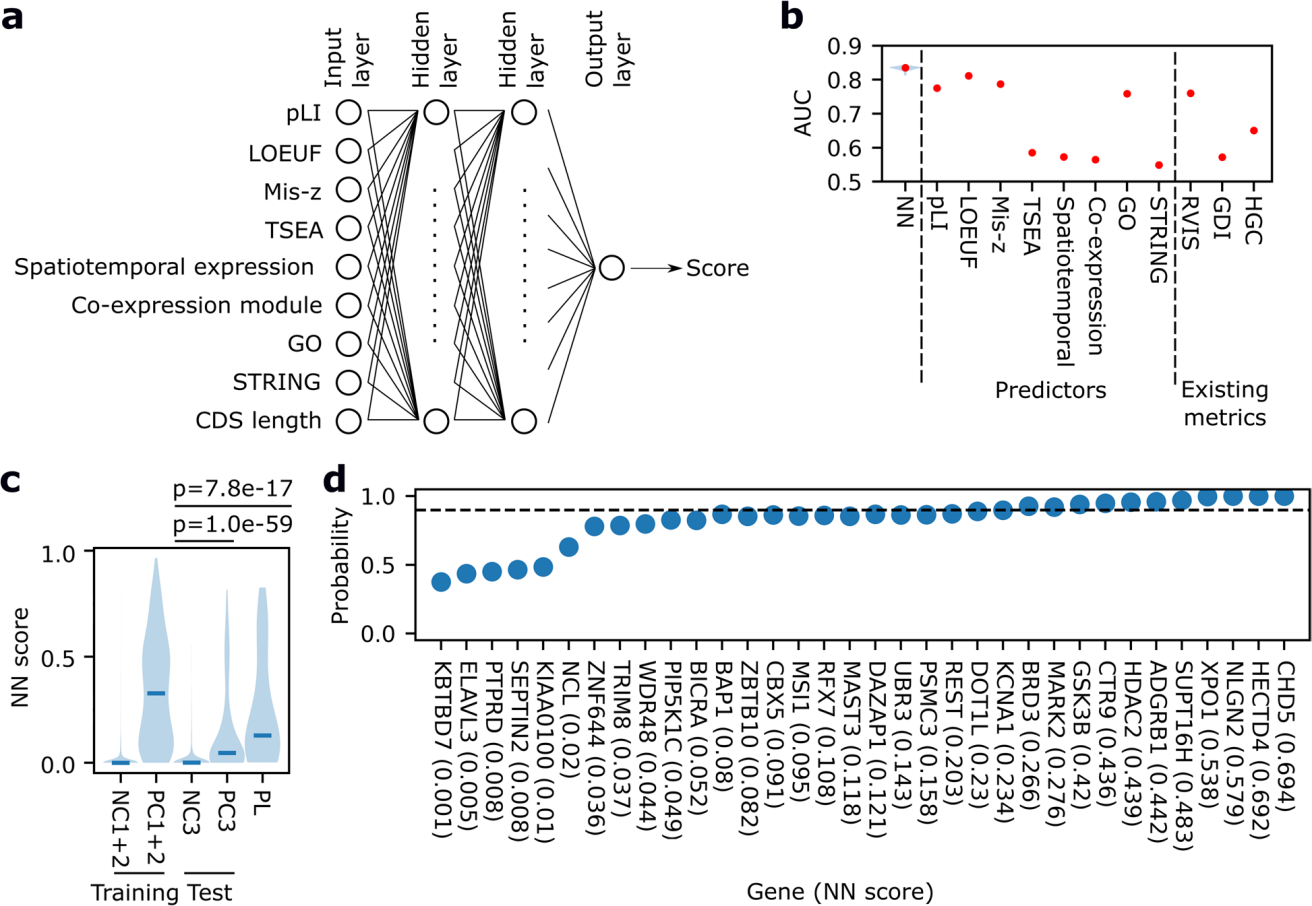
Integration of the bioinformatic analysis results using deep learning. **a** Scheme for the NN model. White circle: neurons of layers; line: connections between neurons. **b** AUC of the full NN model, the eight predictors, and the three existing gene prioritization metrics for PC3 and NC3. The blue violin plot for the NN model (“NN”) represents the distribution based on 500 full NN models, with a red dot indicating the median. **c** Violin plots of the full NN model scores of various gene sets. PL: the 34 plausible candidate genes. *P*-values of one-tailed Wilcoxon rank-sum tests are shown above. **d** Posterior probabilities that the 34 plausible candidate genes are true NDD-associated genes. The probabilities are the median of probabilities computed by 100 full NN models. NN model scores are shown in parentheses. Genes are arranged in the order of NN model scores. Dotted line: 90%

These bioinformatic analyses comparing the 328 known and 34 plausible candidate genes collectively support the relevance of the 34 plausible candidate genes in the pathogenesis of NDDs. Lastly, we sought to construct a model enabling the prioritization of the 34 plausible candidate genes by integrating various information about them. For this purpose, we constructed an NN model estimating the similarity of an input gene to the 328 known genes using the following eight predictors: pLI, LOEUF, missense z-score, and the results of TSEA, brain subregion/stage-specific expression analysis, co-expression module analysis, and STRING analysis (Fig. 6a). In addition to these predictors, we used coding sequence (CDS) length as another input feature, which may affect pLI and LOEUF, such that the model could control for the effect. To accurately train and evaluate this model, we only used genes for which all of the eight predictors were available. We initially trained a model using 243 randomly selected genes of the 328 known genes as positive controls (PC1) and 10,164 non-NDD genes as negative controls (NC1). We then evaluated its performance using the remaining known genes (*n* = 33) and 1124 non-NDD genes (NC2). The model outputs a score for a given gene, and a higher score indicates that the gene is more similar to the 243 known genes. We observed that the scores of the PC2 genes were much higher than those of the NC2 (one-sided Wilcoxon rank-sum test *p*-value = 6.8e-20), and the score distribution of the PC2 was comparable to that of the PC1 (Fig. S9), confirming that the training worked well and the hyperparameter setting prevented overfitting towards the training genes. We then trained the NN model using all the 276 genes and compared this full model with the eight predictors above and three metrics for disease gene prioritization: RVIS, GDI, and HGC [37–40]. Our full NN model had significantly larger AUC for the classification of 246 NDD genes in DDG2P (PC3) and 1000 non-NDD genes (NC3), which are independent of PC1-2 and NC1-2, than the predictors or existing metrics (q-value = 0.010 for LOEUF and 0.0023 for the other predictors and metrics, see “Methods”) (Fig. 6b), indicating that our NN model outperformed the others.

Next, we applied this model to the 34 plausible candidate genes. We found that their scores were also much higher than those of the NC3 (one-sided Wilcoxon rank-sum test *p*-value = 7.8e-17), confirming the overall validity of the 34 plausible genes as candidate NDD genes. On the basis of the obtained scores, we estimated the probabilities that each of the 34 genes is a true-positive NDD gene using the naïve Bayes algorithm. The algorithm calculates posterior probabilities using prior probabilities and likelihood ratios of predictors. We set the prior probabilities that each of the 34 genes is true positive as 63% (33/52), assuming that (1) of the 380 genes with FDR < 0.05, 19 (380 × 0.05) are false positive, (2) the 19 genes are included in the 52 (380−328) new candidate genes whose association with NDD is not known, and (3) the remaining 33 (52−19) genes are true positives. The likelihood ratios of NN model scores were calculated from the score distributions of the PC3 and NC3 (Fig. 6c). Using these prior probabilities and likelihood ratios, we calculated posterior probabilities of the 34 plausible candidate genes (Fig. 6d and Additional file 2: Table S15). We found that 11 out of the 34 genes—*HECTD4*, *CHD5*, *NLGN2*, *XPO1*, *SUPT16H*, *ADGRB1*, *CTR9*, *HDAC2*, *BRD3*, *MARK2*, and *GSK3B*—had > 90% posterior probabilities. Thus, it is considered that these genes in particular are highly likely to be true-positive NDD genes.

## Discussion

In this study, we conducted multi-layered statistical and bioinformatic analyses and achieved the large-scale discovery of novel NDD candidate genes with different levels of confidence. First, we developed a statistical method to analyze dnSNVs and dnCNVs in a uniform framework and applied this to the combined dataset of dnSNVs in 41,165 individuals and dnCNVs in 3675 individuals, including the data newly generated in this study (i.e., the YCU dataset). The analysis identified a total of 52 candidates for novel NDD-associated genes, and dnCNVs contributed to the higher statistical significance for 31 of them, including three novel ones. We next narrowed down the list of candidate genes based on their constraint for deleterious variants in the general population and obtained 34 plausible genes. The overall validity of the 34 genes as NDD genes was supported by multiple lines of evidence from bioinformatic analyses. Lastly, we integrated the results of bioinformatic analyses by constructing a deep learning model and identified 11 genes with > 90% true-positive probability. Besides, we found that many of our new candidates have family genes that are known to be associated with NDDs (e.g., *CHD2*, *CHD3*, *CHD4*, *CHD7*, and *CHD8* for *CHD5*; *HDAC1*, *HDAC3*, and *HDAC8* for *HDAC2*; *KCNA2*, *KCNA4*, *KCNC1*, and *KCNC3* for *KCNA1*; *NLGN3* and *NLGN4X* for *NLGN2*; and *TCF4* and *TCF12* for *TCF3*). Given the usefulness of gene family information in the identification of NDD genes [56], these candidates are considered as true NDD genes with a high level of certainty.

The method uniformly analyzing dnSNVs and dnCNVs that we developed here can be flexibly used in future studies and should accelerate new gene discovery. Indeed, the consideration of dnCNVs in addition to dnSNVs provides a clear advantage as follows. In our model, the number of genes affected by < 1 Mb LOF CNVs per individual is 0.015 (Additional file 2: Table S3) and the number of genes affected by LOF dnSNVs in the trinucleotide context model is 0.085. Therefore, when we analyze dnCNVs together with dnSNVs, we can theoretically gain 18% (0.015/0.085) more de novo LOF variants. Despite dnCNV data being available in a limited number of cases in this study, the addition of the dnCNV data contributed to the discovery of three new genes (*GLTSCR1*, *MARK2*, and *UBR3*). Considering these findings, applying our method to upcoming massive sequencing data obtained in future studies should be beneficial.

The list of dnSNVs and dnCNVs that we compiled can be reusable. We have provided the full list of de novo variants in YCU data and de novo deletions in SSC data (Additional file 2: Table S4, S5, and S6), and this information can be useful in any DNM enrichment analyses. Such future studies may show robust enrichment of damaging DNMs in genes with marginal significance in this study, such as the 18 candidates that we excluded from the 52 candidates when we selected the 34 plausible ones.

The deep learning model that we have developed can objectively quantify how functionally valid a new candidate gene is from multiple types of and sometimes redundant information. Given the highly and easily customizable nature of this model, it may also be effective in prioritizing new candidate genes of other diseases based on any predictors though the accuracy depends on the number of known genes responsible for the diseases. While in this study, we used this model to identify better candidates from a limited number of selected genes, and it also enables ab initio identification of good candidates from among all genes. For example, some of the genes that we considered as negative control genes, whose association with NDDs has never been reported, actually showed high scores comparable to those of known NDD genes (Additional file 2: Table S15). These genes may be good candidates for new NDD-associated genes.

Regarding the limitations of our study, first, the sizes of the dataset of CNVs used for construction and validation of the model predicting dnCNV rates are still insufficient (Additional file 1: Fig. S1d, f, and h), although we used the largest available datasets, such as those from gnomAD-SV and SSC. Thus, larger data of SVs in populations are awaited. Second, for simplicity, in this study, we considered LOF-tolerant genes flanked by 1 Mb upstream and downstream regions without any LOF-intolerant genes as being “neutral” and used them for the model training. However, this method does not account for the possibility that there would be functional noncoding elements. By better understanding and integrating the information of such elements, we would be able to further improve the accuracy of the model. Third, in our gene-based enrichment analysis, we discarded the calls of dnCNVs larger than 1 Mb, which were observed in 2.1% (27/1298) of YCU and 0.59% (14/2377) of SSC probands. Most of these large dnCNVs are likely pathogenic in light of their very-low frequencies in SSC healthy siblings, that is, 0.052% (1/1922). Although we did not use the data of such large dnCNVs in our current study considering that the majority of dnCNVs larger than 1 Mb overlapped with two or more LOF-intolerant (pLI > 0.9 or LOEUF < 0.35) genes (83% and 79% in YCU and SSC probands, respectively), we would be able to identify additional NDD genes by efficiently incorporating the data of large dnCNVs. Fourth, we may underestimate the true-positive probabilities of the 34 plausible candidate genes (Fig. 6d) because positive controls used for the model construction (PC1-2) and those for the likelihood ratio calculation (PC3) had different characteristics. The PC1-2 and PC3 had similar but slightly different distributions of NN model scores (Fig. 6c). The reason for this may be that the PC1 could be biased due to the detection method, that is, our DNM enrichment analysis method, which is less capable of detecting genes with pathogenic missense variants than those with pathogenic LOF variants because of difficulty in annotating the pathogenicity of missense variants. The lower NN model scores in the PC3 led to underestimation of the likelihood ratios and the resulting posterior probabilities of the 34 candidate genes (Fig. 6c). Therefore, we expect that the 34 candidate genes may be more likely to be true positives than estimated by this model. Lastly, while we focused only on DNMs, other inheritance patterns (e.g., autosomal recessive) can be involved in the genetic risks of NDDs. Future works considering genes underlying NDDs of these inheritance modes should be fruitful.

## Conclusions

To identify new NDD-associated genes, we developed a method that evaluates the burdens of dnSNVs and dnCNVs in a uniform framework and compiled comprehensive lists of dnSNVs and dnCNVs by aggregating data from our own new dataset and published studies. Leveraging these improvements, we identified a large number of new candidate genes. From these candidates, we obtained more than 10 genes with high true-positive probabilities using deep learning. These new genes should contribute to further elucidation of the genetic architecture of NDDs, and the methods and resources that we developed here can be used in future studies to identify more NDD-associated genes.

## Supplementary information

**Additional file 1. **Supplementary Methods, Results and Figures. **Figure S1.** Calculation of mutation rates of < 1 Mb LOF CNVs per gene. **Figure S2.** Analyses of CNVs in WGS data of SSC ASD quads. **Figure S3.** CNV QC of YCU WES data. **Figure S4.** Comparison of the observed and expected numbers of DNMs in YCU, DDD31k, and denovo-db data. **Figure S5.** dnCNVs at the 380 DNM-enriched genes in SSC data. **Figure S6.** dnCNVs at the 380 DNM-enriched genes in YCU data. **Figure S7.** Plots of DNMs at the 52 DNM-enriched candidate genes. **Figure S8.** Enrichment of constrained genes in the 52 DNM-enriched candidate genes. **Figure S9.** Distributions of NN model scores in NC1 and PC1 training and NC2 and PC2 test gene sets.

**Additional file 2: Table S1.** Summary of explaining and response variables in the regression analyses. **Table S2.** Summary of models for relative number of < 1 Mb LOF CNV per gene. **Table S3.** Mutation rate of < 1 Mb LOF CNV per gene. **Table S4.** Rare de novo variants in 1,317 YCU samples. **Table S5.** Calls of < 1 Mb rare de novo deletions in 1,298 YCU samples. **Table S6.** Calls of < 1 Mb rare de novo deletions in 2,377 SSC probands. **Table S7.** Results of DNM enrichment analyses in the DNM-enriched 381 genes including *ATP6V0C*. **Table S8.** DNMs at the 381 DNM-enriched genes including *ATP6V0C*. **Table S9.** Confidence and predicted pathomechanism of in the 52 new DNM-enriched genes. **Table S10.** TSEA of the 328 known and 34 plausible candidate genes. **Table S11.** Enrichment analyses of genes specifically expressed in various brain regions and developmental stages in the 328 known and 34 plausible candidate genes. **Table S12.** Enrichment analyses of genes specifically expressed in co-expression modules in the 328 known and 34 plausible candidate genes. **Table S13.** Enrichment analyses of GO terms in the 328 known and 34 plausible candidate genes. **Table S14.** Enrichment analyses of proteins of STRING clusters in the proteins encoded by the 328 known and 34 plausible candidate genes. **Table S15.** Inputs and results of deep learning analysis.

## Data Availability

The datasets analyzed and/or generated during the current study are available: the list of CNVs in gnomAD-SV: https://gnomad.broadinstitute.org/downloads/gnomad_v2_sv.sites.bed.gz [15]; pLI, LOEUF, missense z-score, and o/e ratio of LOF SNVs in gnomAD: https://gnomad.broadinstitute.org/downloads/gnomad.v2.1.1.lof_metrics.by_transcript.txt.bgz [19]; denovo-db: https://denovo-db.gs.washington.edu/denovo-db/Download.jsp [10]; de novo variants in DDD31k: https://www.nature.com/articles/s41586-020-2832-5 [1]; de novo variants and deletions in YCU: Additional file 2: Table S4 and S5; de novo deletions in SSC: Additional file 2: Table S6; per-gene mutation rates of < 1 Mb LOF CNVs: Additional file 2: Table S3; deletion intolerance score in ExAC: gs://gcp-public-data—gnomad/legacy/exacv1_downloads/release0.3.1/cnv [43]; dosage sensitivity genes: https://ftp.clinicalgenome.org/ClinGen_gene_curation_list_GRCh38.tsv [53]; the lists of genes expressed specifically in brain spatiotemporal coordinates: http://genetics.wustl.edu/jdlab/psi_package/pSI.data_1.0.tar_.gz/data/human.rda [30]; GDI score: https://lab.rockefeller.edu/casanova/GDI/GDI_full.txt [38]; The HGC server: https://hgc.rockefeller.edu/ [40]; RVIS: http://genic-intolerance.org/data/RVIS_Unpublished_ExACv2_March2017.txt [37]; codes: https://github.com/hamanakakohei/GenomeMed2022/ [20].

## Abbreviations

NDD: Neurodevelopmental disorder
CNV: Copy number variant
SNV: Single-nucleotide variant
FDR: False discovery rate
WES: Whole-exome sequencing
DNM: De novo mutation
dn: De novo
SD: Segmental duplications
LOF: Loss-of-function
ExAC: Exome Aggregation Consortium
gnomAD: Genome Aggregation Database
gnomAD-SV: gnomAD-structural variation
LOEUF: Loss-of-function observed/expected upper bound fraction
AIC: Akaike’s information criterion
SSC: Simons Simplex Collection
YCU: Yokohama City University
DDD: Deciphering Developmental Disorders project
MPC: Missense badness, PolyPhen-2, and Constraint
BH: Benjamini Hochberg
qPCR: Quantitative polymerase chain reaction
IGV: Integrative Genomics Viewer
DDG2P: Development Disorder Genotype Phenotype Database
GTEx: Genotype-Tissue Expression
pSI: Specificity index probability
M: Module
BP: Biological process
CC: Cellular component
MF: Molecular function
PPI: Protein–protein interaction
NN: Neural network
TSEA: Tissue-specific expression analysis
PC: Positive control
NC: Negative control
ASD: Autism spectrum disorder
