# Supplementary material for: Large-scale discovery of novel neurodevelopmental disorder-related genes through a unified analysis of single-nucleotide and copy number variants
Source: Genome Med. 2022 Apr 26;14:40. doi: 10.1186/s13073-022-01042-w (PMC9040275; doi:10.1186/s13073-022-01042-w)
Supplement: Supplementary file 1 — Additional file 1. Supplementary Methods, Results and Figures. Figure S1. Calculation of mutation rates of < 1 Mb LOF CNVs per gene. Figure S2. Analyses of CNVs in WGS data of SSC ASD quads. Figure S3. CNV QC of YCU WES data. Figure S4. Comparison of the observed and expected numbers of DNMs in YCU, DDD31k, and denovo-db data. Figure S5. dnCNVs at the 380 DNM-enriched genes in SSC data. Figure S6. dnCNVs at the 380 DNM-enriched genes in YCU data. Figure S7. Plots of DNMs at the 52 DNM-enriched candidate genes. Figure S8. Enrichment of constrained genes in the 52 DNM-enriched candidate genes. Figure S9. Distributions of NN model scores in NC1 and PC1 training and NC2 and PC2 test gene sets. [file 13073_2022_1042_MOESM1_ESM.docx]

**Contents**

Supplementary Methods………………………..………………………………………. 2

Supplementary Results…………………………………………………………………. 6

Supplementary Figures…………….…………………………………………………... 10

Figure S1. Calculation of mutation rates of < 1 Mb LOF CNVs per gene………........ 10

Figure S2. Analyses of CNVs in WGS data of SSC ASD quads…………………….. 13

Figure S3. CNV QC of YCU WES data…………………………………………........ 14

Figure S4. Comparison of the observed and expected numbers of DNMs in YCU, DDD31k, and denovo-db data…………………………………………………...…... 15

Figure S5. dnCNVs at the 380 DNM-enriched genes in SSC data………………….. 16

Figure S6. dnCNVs at the 380 DNM-enriched genes in YCU data…………………. 20

Figure S7. Plots of DNMs at the 52 DNM-enriched candidate genes……………….. 24

Figure S8. Enrichment of constrained genes in the 52 DNM-enriched candidate genes…………………………………………………………………………………. 26

Figure S9. Distributions of NN model scores in NC1 and PC1 training and NC2 and PC2 test gene sets……………………………………………………………………. 27

References………………………………………...…………………........................... 28

**Supplementary Methods**

**Analysis of de novo copy number variants (dnCNVs) in whole-genome sequencing (WGS) of Simons Simplex Collection (SSC) autism spectrum disorder (ASD) quads**

We downloaded variant call formant (VCF) files (GS.sv.genotype.filtered.vcf, hg38) containing deletion CNVs in quads with one child affected by ASD and one or more unaffected siblings from SSC. When a family had more than one unaffected sibling, we selected only one sibling (named s1). CNVs were discovered with GenomeSTRiP v2.00 by the data provider. We discarded CNVs with no data on genotype quality (GQ) or GQ lower than a threshold, which we varied from 0 to 90, in any of the trio members. To analyze the sensitivity of the GenomeSTRiP analysis, we used deletion CNVs discovered in a subset (n = 519) of the families with the Genome Aggregation Database structural variation (gnomAD-SV) pipeline and confirmed by quantitative polymerase chain reaction (qPCR) in a previous study.^1^ CNVs identified in de novo mutation (DNM)-enrichment analyses were subsequently confirmed using Integrative Genomic Viewer (IGV) [1].

**Sample and variant quality check (QC) in Yokohama City University (YCU) whole-exome sequencing (WES) data**

WES was performed for 13,851 samples including 2536 trios, as previously described [2]. In brief, genomic DNA was captured with a SureSelect Human All Exon V4, V5, or V6 Kit (Agilent Technologies, Santa Clara, CA, USA). Sequencing was performed on a HiSeq 2500 (Illumina, San Diego, CA, USA) with 101 bp paired-end reads. Reads were aligned to the human reference genome (GRCh37.1/hg19) using Novoalign v3.02.13. Polymerase chain reaction (PCR) duplicates were removed using Picard. Local realignments around indels and base quality score recalibration were performed with the Genome Analysis Toolkit (GATK) 3.7-0, and analysis-ready binary alignment map (BAM) files were generated [3]. From the BAM files, variants were jointly genotyped with GATK HaplotypeCaller and annotated with the variant quality score recalibration (VQSR) score following GATK Best Practice [4]. The functional consequences of the variants were annotated using snpEff [5]. We obtained several metrics using GATK VariantEval: number of single-nucleotide variants (SNVs), number of insertions, number of deletions, the ratio of insertions to deletions, the ratio of heterozygous to homozygous variants, and the ratio of transitions to transversions in each sample. We removed outlier individuals for either of the metrics.

To confirm chromosomal sex in each sample as described previously [6], we manipulated the VCF file using GATK SelectVariants, GATK VariantFiltration, and vcftools as follows: 1) selected variants at chr.X or Y but not at pseudoautosomal regions; 2) regarded variants with read depth of < 10 in each sample as missing; and 3) selected variants whose missing rate was < 0.05 and minor allele frequency (MAF) in the VCF file was > 0.01. We analyzed these filtered variants using the check-sex option of PLINK and obtained inbreeding coefficients (F) at chr.X and variant count at chr.Y. Plotting the F at chr.X and variant count at chr.Y showed two clusters of male and female individuals (data not shown). From this plot, we removed individuals who were outliers from the two clusters.

To analyze relatedness among samples as previously described [6], we extracted variants using PLINK as follows: 1) > 10% MAF in the VCF; 2) < 10% missingness; 3) p-value > 0.05 in Hardy–Weinberg equilibrium test; 4) biallelic; and 5) not in linkage disequilibrium with > 2 variance inflation factors at every 50 variants. On the basis of these extracted variants, we excluded individuals for whom > 10% variants were missed using PLINK. For the remaining variants in the remaining samples, we obtained PIHAT and the probability that there was no identity by descent [P(IBD=0)] among samples using the PLINK –genome option. Plotting PIHAT and P(IBD=0) for each pair (n = _13,851_C_2_), we removed pairs that were outliers from a cluster of child–parent pairs.

To analyze sample contamination as previously described [6], we used variants filtered in the relatedness analysis. We calculated F and means of PIHATs to all other samples in each sample using the PLINK –het option. We regarded outlier samples with low F and high mean of PIHAT as being contaminated and removed them.

**Filtering de novo SNVs and small indels and additional QC of samples in YCU WES data**

We filtered variants as previously described [7]: 1) MAF < 0.001% in 5575 healthy YCU samples and the “non-neuro” subset of Genome Aggregation Database (gnomAD); 2) called by GATK in the case but not in the parents; and 3) > −29.2 and > −7.58 variant quality score log odds (VQSLOD) scores for SNVs and small indels, respectively, > 5.5 Triodenovo scores for SNVs and small indels [8], > 0.25 DNMfilter score for SNVs [9], and > 0.005 Denovogear score for SNVs [10]. After such filtering, we removed samples with an outlying number (> 9) of DNMs.

**Filtering dnCNVs and additional QC of samples in YCU WES data**

To detect CNVs, we joint-genotyped 8696 samples using eXome Hidden Markov Model (XHMM), a subset of the above 13,851 samples, including all the 2536 YCU trios and additional cases having CNVs and healthy controls as previously described [11, 12, 13]. The analysis outputted an XCNV file containing CNV calls in each sample and a VCF file containing Q_SOME (SQ), a quality metric for all CNV called regions × all samples. We analyzed only CNVs overlapping with > 3 baits. We regarded CNVs whose SQ was > 90 in the proband and < 90 in the parents of each trio as dnCNVs. We regarded CNVs whose SQ was > 90 in < 3 individuals among 5566 healthy individuals as rare. In addition to the above sample QC using SNVs, we excluded samples meeting at least one of the following criteria to perform additional sample QC: samples with > 20 deletions, > 20 duplications, > 30 deletions and duplications, > 10 de novo deletions, > 10 de novo duplications, > 15 de novo deletions and duplications, > 3 rare de novo deletions, > 3 rare de novo duplications, and/or > 4 rare de novo deletions and duplications.

**Case reports**

We described the clinical details of two cases with a de novo variant in *PIP5K1C* (c.662A>G). One case was among the YCU samples included in the DNM enrichment analyses (n=1317) while the other case was later recruited at YCU for genetic diagnosis, whose *PIP5K1C* variant was detected by WES. The clinical details were collected by attending physicians.

**Statistics**

To model the number of < 1 Mb loss-of-function (LOF) CNVs per gene in gnomAD-SV, we performed negative binomial regression analyses because the number of CNVs per gene is count data with no upper limit. To analyze the enrichment of DNMs, we performed Poisson tests based on the previous finding that the number of mutation events in each individual follows a Poisson distribution [14].

**Supplementary Results**

**Sample and dnCNV QC in WGS of SSC ASD quads**

To perform sample QC, we counted deletion dnCNVs in ASD probands and healthy siblings. We found that several probands (8/2385) and healthy siblings (6/1928) had an outlying number (> 10) of dnCNVs (Additional file 1: Fig. S2a). We removed these individuals in the following analyses. Next, to optimize dnCNV detection in the SSC pipeline that uses GenomSTRiP, we optimized the threshold of GQ score for CNV calling using three metrics: 1) sensitivity to positive control deletion dnCNVs experimentally validated in a previous study [15]; 2) the number of detected deletion dnCNVs; and 3) the transmission rate of deletion CNVs from the parents (Additional file 1: Fig. S2b–d). The sensitivity of the SSC pipeline to the positive control dnCNVs was 59.7% (46/77) when there was no filtering of CNVs (GQ = 0). The region called by the GenomeSTRiP pipeline > 90% mutually overlapped with the region experimentally validated, except for one dnCNV. The sensitivity of the SSC pipeline was not high because the positive control dnCNVs were detected using a highly sensitive method combining multiple software programs in previous research.^1^ Most of the missed CNVs were less than 1 kb long, suggesting low sensitivity of GenomeSTRiP for CNVs of this size (Additional file 1: Fig. S2e). When the GQ threshold is more stringent, the sensitivity is lower, the number of deletion dnCNVs is smaller, and the rates of transmission of deletion CNVs in parents to probands and siblings are closer to 50%, the theoretical rate (Additional file 1: Fig. S2b–d). When the GQ threshold was set to 40, the three metrics are well balanced as follows: sensitivity = 59.7%, per-individual deletion dnCNVs in probands = 0.211 (501 dnCNVs in 2377 probands), per-individual deletion dnCNVs in siblings = 0.151 (290 dnCNVs in 1922 siblings), and rates of transmission of deletion CNVs in parents to probands and siblings = 49.7% and 49.7%, respectively. After these sample- and variant-level QCs, 89 rare de novo LOF deletions in 2377 probands in SSC quads were available for DNM enrichment analyses (Additional file 2: Table S6).

**CNV QC and additional sample QC of YCU WES data**

To detect CNVs, we joint-genotyped all the 2536 trios and additional cases having CNVs and healthy controls (8696 samples in total) using XHMM to establish the variant filtering strategy as shown below and detect rare dnCNVs for DNM enrichment analyses. To filter the CNVs, we optimized the threshold for Q_SOME (SQ), a quality metric of CNVs, using qPCR-confirmed CNVs: 34 deletions (44 calls) and 19 duplications (32 calls). We compared the SQ of each CNV region between the carrier and control individuals (n = 5468), which excluded the family members of the carriers (Additional file 1: Fig. S3). From the distributions of SQ, we set the threshold as 90, and the sensitivity was 100% (44/44) for deletions and 97% (31/32) for duplications. The sensitivity was overestimated because most of the deletions and duplications had been previously identified with XHMM. Several CNVs had SQ of > 90 in some control individuals. Some CNVs were divided and called as multiple smaller CNVs based on highly variable coverage, especially at segmental duplication (SD) regions in the centromere, subtelomere, or 16p13.11. Among the smaller CNVs, CNVs containing the SDs (chr1:1,421,483–1,444,865, chr1:1,583,363–1,635,875, chr16:14,916,714–15,083,999, and chr21:10,908,792–10,998,340) had SQ of > 90 in many control individuals and might be false positive. By contrast, most of the other CNV calls with SQ of > 90 in control individuals were likely true positive based on the manual inspection of coverage. It was supposed that the pathogenic CNVs were observed in control individuals for the following reasons: the CNVs were not fully penetrant (chr2:111,395,546–113,090,073: 2q13 deletion syndrome; chr16:14,916,714–15,083,999, chr16:15,123,812–15,141,893, chr16:15,125,634–16,315,604, chr16:15,125,634–16,315,604: 16p13.11 deletion syndrome; chr16:15,494,660–17,451,968: 16p13.11 duplication syndrome; chr16:29,610,505–30,217,589: 16p11.2 duplication syndrome; chr22:18,893,960–21,411,567: 22q11 duplication syndrome) or the CNVs were tolerable in females (chrX:153,030,920–153,609,603 and chrX:153,055,060–153,609,603). Thus, the SQ threshold had high sensitivity and specificity, except for regions overlapping with SDs. After we filtered CNVs with the SQ threshold and samples with outlying numbers of CNVs (see “*Filtering of dnCNVs and additional QC of samples in YCU WES data*”), the mean numbers of deletions, duplications, and all rare dnCNVs per sample were 0.10, 0.11, and 0.21, respectively, and 114 rare de novo LOF deletions in 1298 neurodevelopmental disorder (NDD) cases remained for DNM enrichment analyses (Additional file 2: Table S5).

**Case report of two YCU individuals showing a de novo missense variant at *PIP5K1C* (Sample_8283 and Sample_26613; chr19:3653547T>C; ENST00000589578: c.662A>G, p.Tyr221Cys)**

Sample_8283: This case involved an 11-year-old girl. From the neonatal period onward, she exhibited intractable epilepsy. Currently, she has tonic seizures a few times a day, severe intellectual disability, facial dysmorphism, and constriction of the upper respiratory tract. She speaks no meaningful words.

Sample_26613: This case involved a girl aged 3 years and 8 months. She had a small head circumference from birth (−2 sd, 32 cm) and showed profound global developmental delay and significant postnatal slowing of head growth. She had recurrent aspiration pneumonia with persistent right upper lobe collapse at 6 months and 9 months of age and was recognized to have grade 3 gastroesophageal reflux from barium swallow. Flexiscope showed laryngopharyngeal reflux. She had frequent spasms, which resolved with clonazepam. Physical exams showed low anterior hairline, anterior cowlick, sparse flared eyebrows, down-slanting and short palpebral fissures, left partial ptosis, large and asymmetrical ears, circumferential skin folds at the forearms and ankles, and extensive Mongolian spots over both lower and upper limbs. Magnetic resonance imaging (MRI) showed cerebral atrophy. Currently, her height is 93 cm (−1 sd), her weight is 9.7 kg (< 3^rd^ percentile), and her head circumference is 42 cm (−7 sd). She still shows head lag. She rolls over but cannot sit unsupported. She can only make incomprehensible sounds and speaks no meaningful words. She has hypotonia.





**Figure S1. Calculation of mutation rates of < 1 Mb LOF CNVs** **per gene**

(a) Correlation between total mutation rate per gene and number of observed variant sites per gene in 10,000 individuals when the variants are under no natural selection in genetic drift simulations. Each dot from left to right in all panels corresponds to the number of mutation sites: 100, 320, or 1000. Mutation rates per mutation site are shown above each panel. Regardless of the parameter settings, the total mutation rates in a supposed gene and the mean numbers of variant sites observed in 10,000 individuals in the last generation in the supposed gene in > 300 simulations were correlated and aligned on the same dashed line. Dashed line: an arbitrary reference line shared in all of the panels. (b) Plots of numbers of < 1 Mb LOF CNV sites per gene observed in gnomAD-SV and six candidates of explanatory variables in the training genes (red plot, upper) and the other genes (blue plot, lower) at autosomes. Pearson’s correlation coefficients and p-values are presented above each plot. The dotted vertical line in the panel of distance from the telomere indicates 25 Mb at the x-axis, before and after which the trend of the correlation changed. Dot: each gene; black line: moving average of the number of < 1 Mb LOF CNV sites. (c) Correlation between the observed number of < 1 Mb LOF CNV sites in gnomAD-SV and the expected number of < 1 Mb LOF CNV sites based on the fourth model (Additional file 2: Table S2) for the training genes (left) and the other genes (right) at autosomes. Note that genes whose observed number was > 10 are not shown to clearly visualize genes with 10 or fewer CNV sites. Pearson’s correlation coefficient and p-value are shown above. Dot: each gene; black dashed line: x = y; red line: moving average. (d) Correlation between the ratios of observed to expected numbers (o/e ratios) of LOF SNV sites in gnomAD and < 1 Mb LOF CNV sites in both the training genes and the other genes at autosomes. The o/e ratios of < 1 Mb LOF CNV sites were based on the fourth model (Additional file 2: Table S2). Dot: each gene; black dashed line: x = y; red line: moving average. (e) Correlation between the o/e ratio of LOF CNV sites in gnomAD-SV and deletion intolerance z-score in Exome Aggregation Consortium (ExAC) among 15,606 autosomal genes [16]. Dot: each gene; red line: regression line. (f) Estimates of the total mutation rate of < 1 Mb LOF dnCNVs at the training genes using Watterson estimator. The total mutation rates calculated from experimentally confirmed < 1 Mb LOF dnCNVs in ASD probands or healthy siblings in 519 SSC quads in a previous study are shown for comparison [15]. Error bar: 95% confidence interval (CI). (g) Numbers of affected training genes per < 1 Mb LOF CNV at training genes in gnomAD-SV. The CNVs were categorized by MAF. Dots: mean; error bar: 95% CI based on the bootstrapping method. (h) The total number of genes affected by < 1 Mb LOF dnCNVs at autosomes, which was calculated as the sum of the absolute mutation rates per gene in autosomal genes (Additional file 2: Table S3). The total numbers of genes affected by < 1 Mb LOF dnCNVs at autosomes in ASD probands or healthy siblings in 519 SSC quads in a previous study are shown [15]. Error bar: 95% CI based on the bootstrapping method.

**
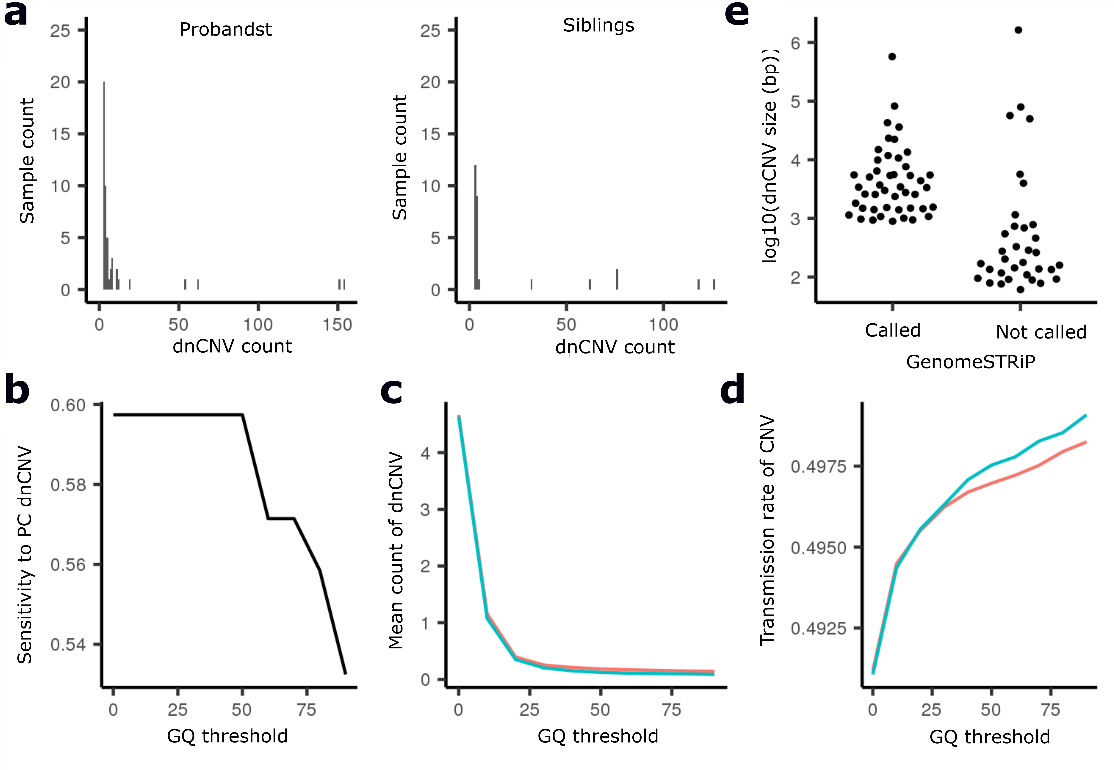
**

**Figure S2**. **Analyses of CNVs in WGS data of SSC ASD quads**

(a) Sample QC using numbers of deletion dnCNVs identified with GenomeSTRiP. Note that the bar of 0 dnCNV count is not shown in the histogram to clearly visualize short bars of > 10 dnCNV counts. (b–d) Optimization of the threshold for genotype quality (GQ) scores of GenomeSTRiP. We plotted sensitivity to positive control dnCNVs (b), numbers of deletion dnCNVs (c), and transmission rates (d) using various thresholds for GQ. Red line: probands; blue line: siblings. PC, positive controls. (e) Sizes of positive control dnCNVs called or not called with GenomeSTRiP.

**
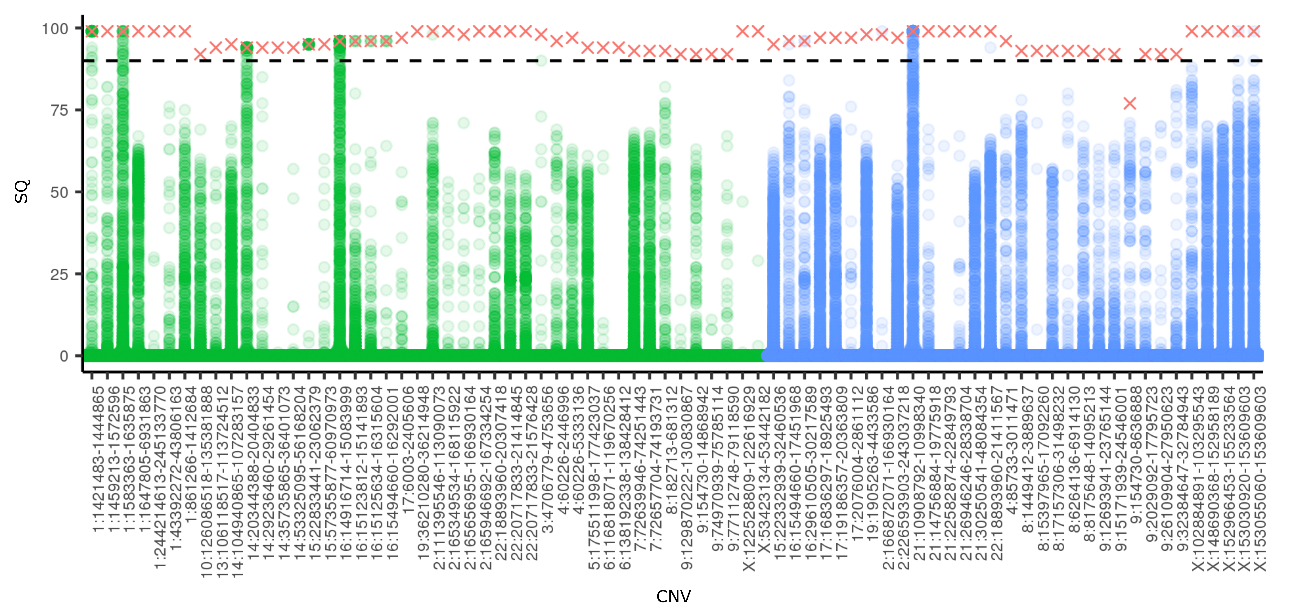
**

**Figure S3**. **CNV QC of YCU WES data**

A plot of SQ scores of qPCR-confirmed CNVs (44 deletion and 32 duplication calls) in the carriers (cross marks) and the corresponding regions in 5468 control individuals (circle marks). The horizontal dashed line indicates SQ = 90. Green mark: deletion; blue mark: duplication.

**
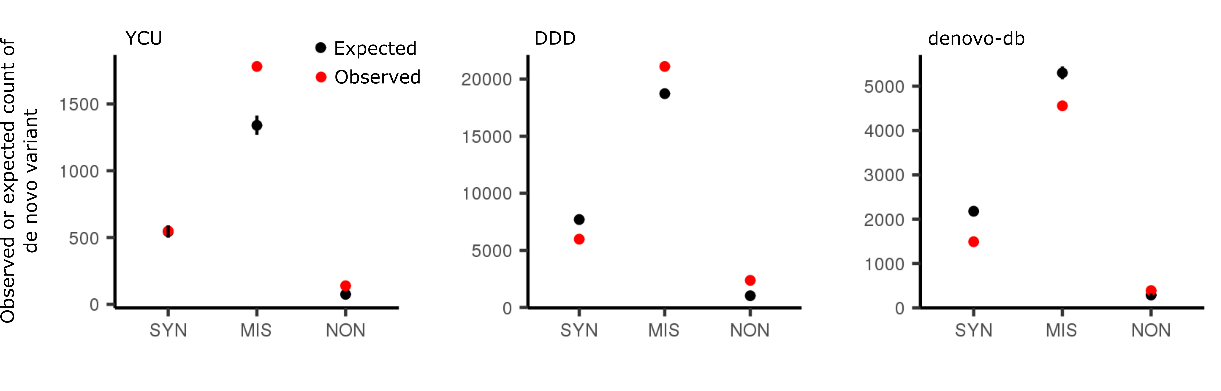
**

**Figure S4**. **Comparison of the observed and expected numbers of DNMs in YCU, DDD31k, and denovo-db data**

We compared the observed (red dots) and expected numbers (black dots) of de novo synonymous (SYN), missense (MIS), or nonsense (NON) variants in YCU (n = 2466), DDD31k (n = 31,058), and denovo-db data (n = 8790). The expected numbers of DNMs in YCU were calculated with an adjustment based on the sequencing depth, while those in DDD31k and denovo-db were obtained without the depth-based adjustment (see Additional file 1: Supplementary Material and Methods). Error bar: 95% CI.


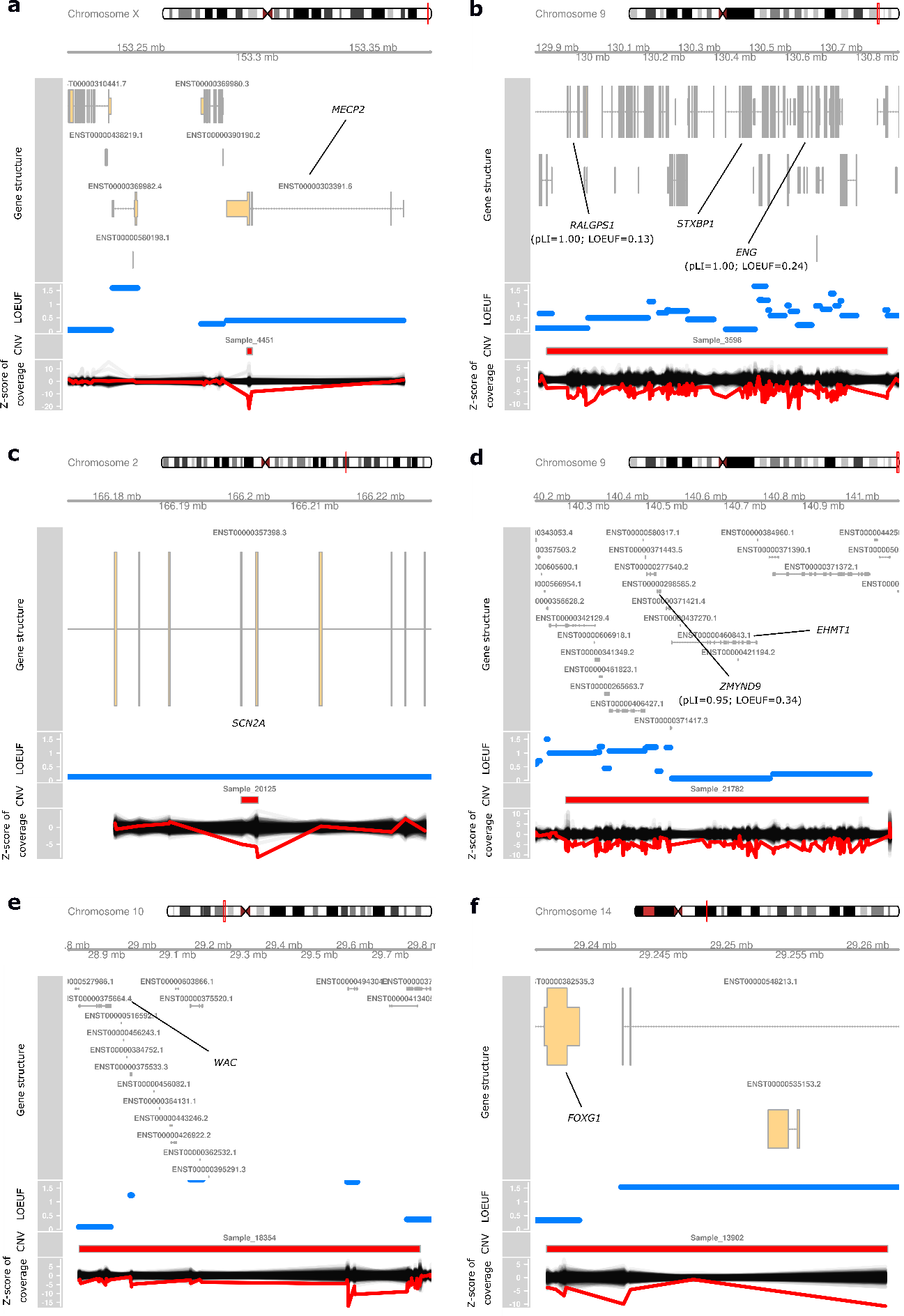


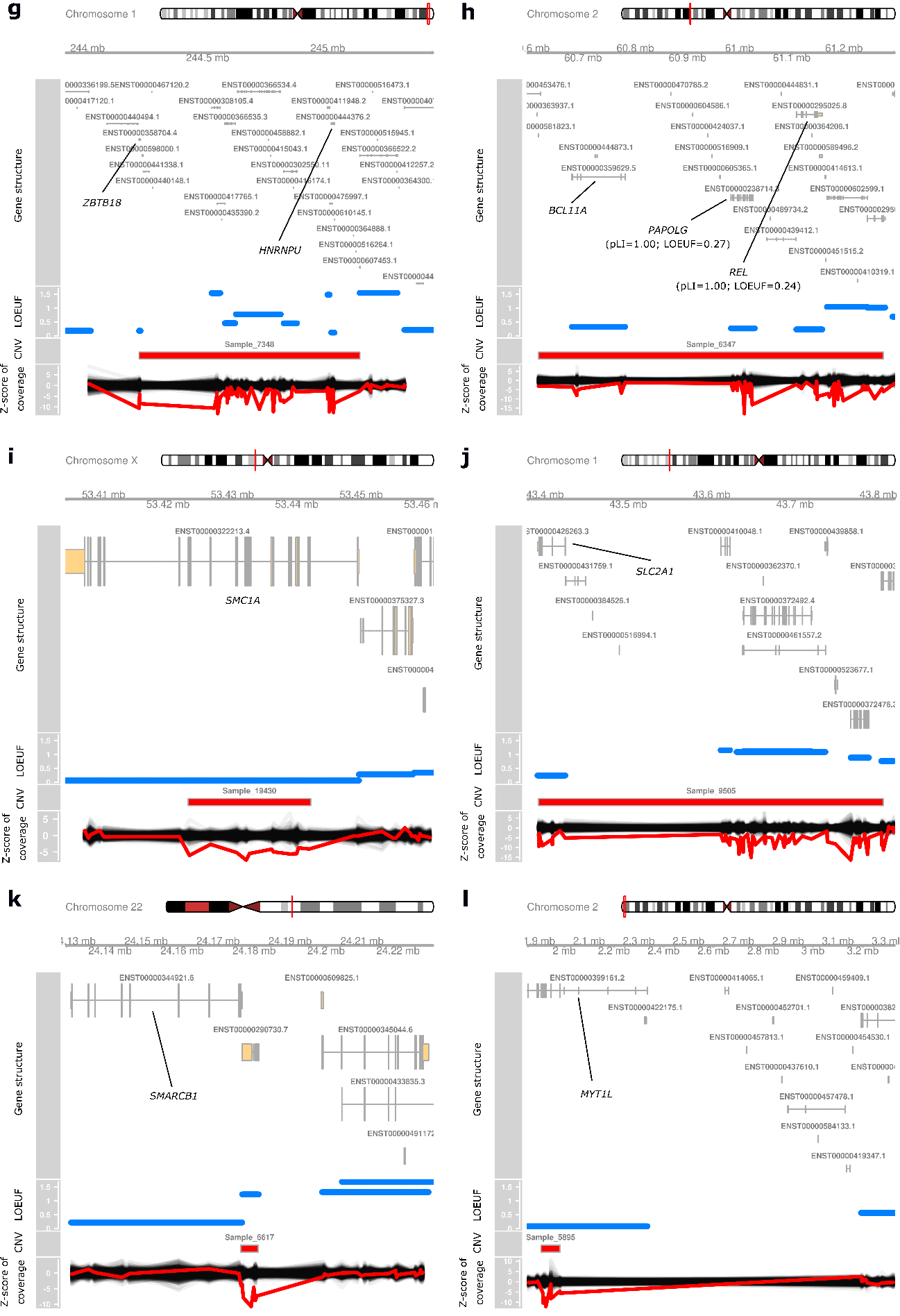


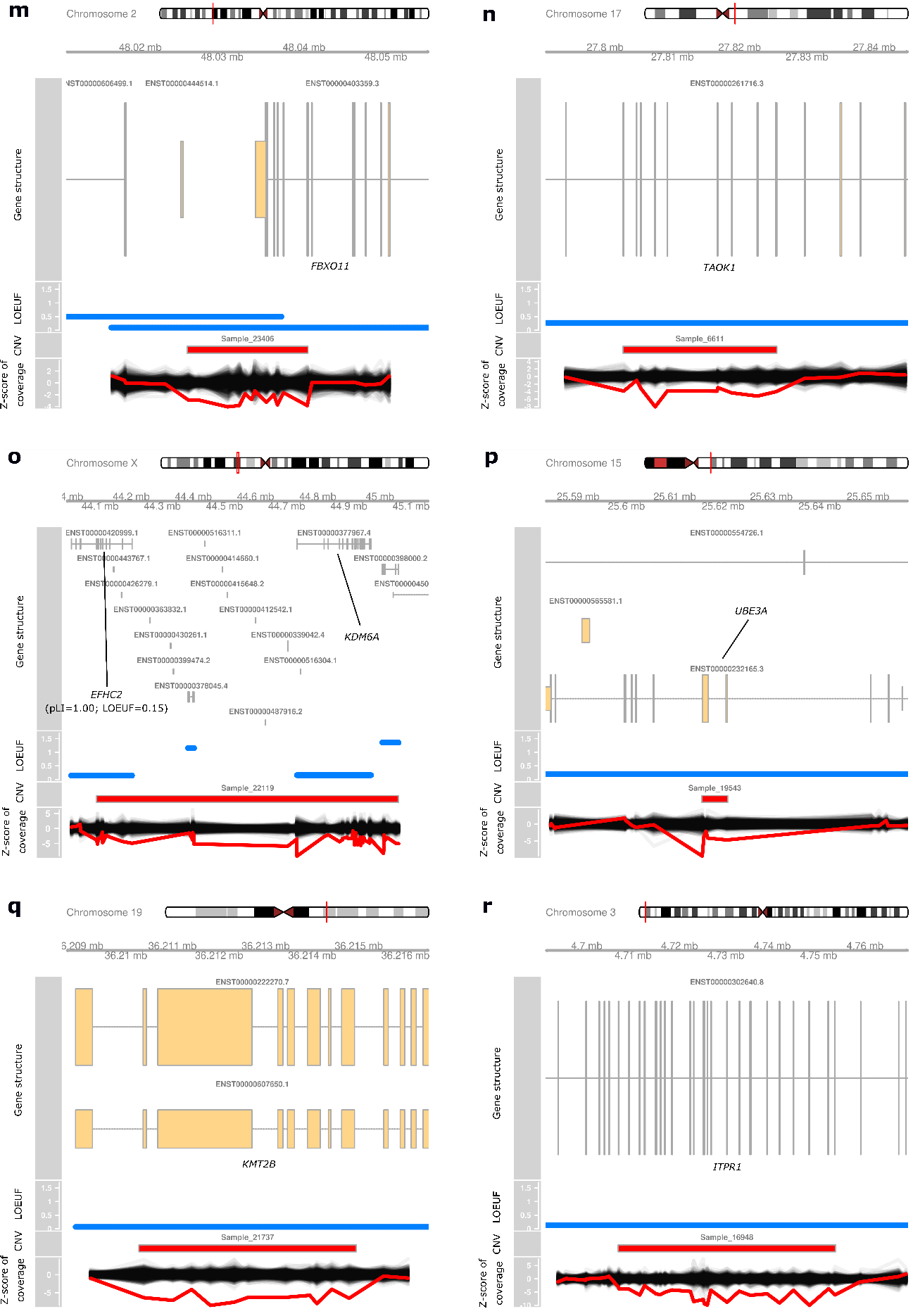


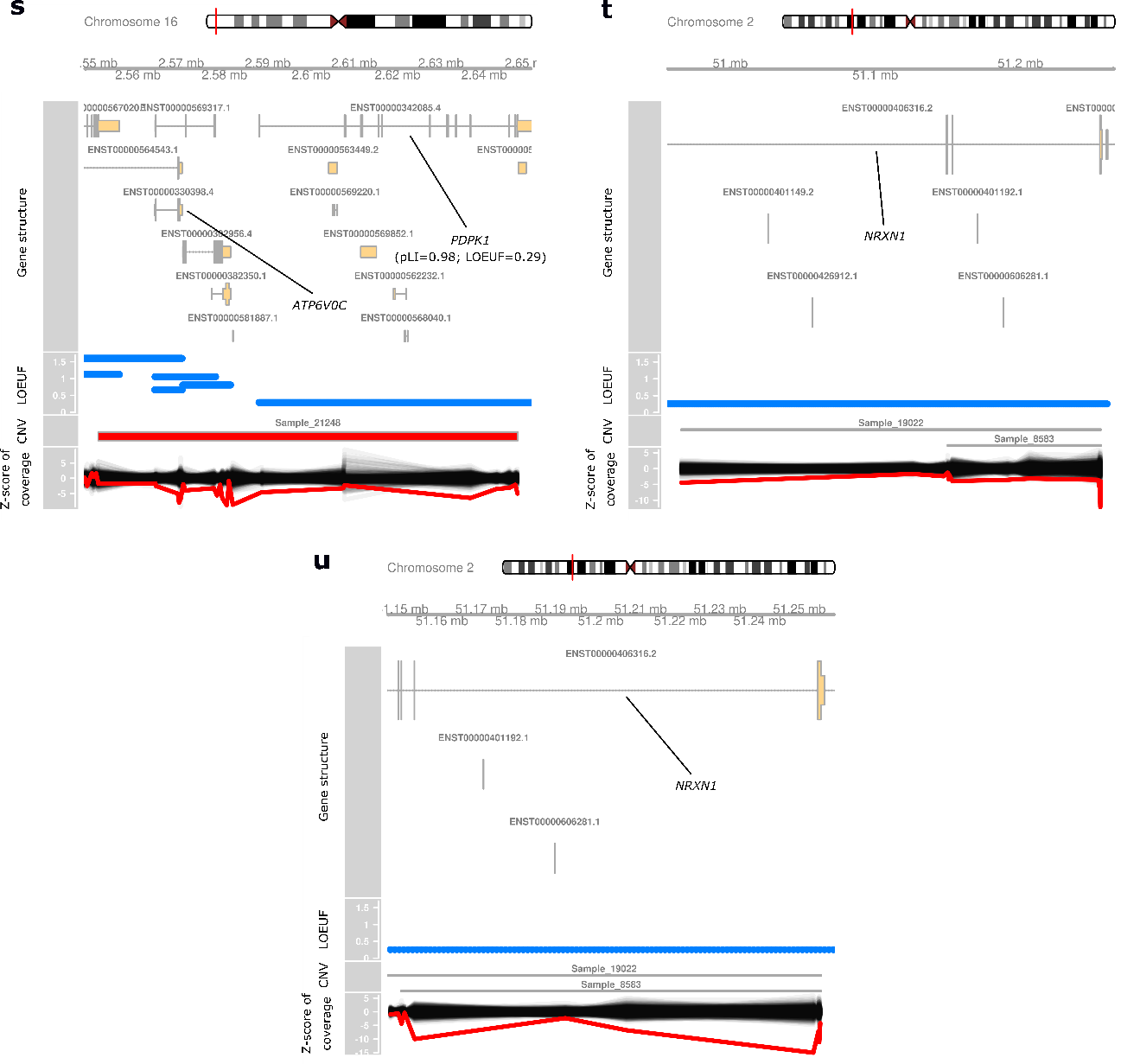


**Figure S5. dnCNVs at the 380 DNM-enriched genes in YCU data**

Visualization of LOF dnCNVs as shown in Fig. 2b. From top to bottom, the plots show the exon–intron structures of the canonical gene transcripts, loss-of-function observed/expected upper bound fraction (LOEUF), CNVs called by XHMM, and z score of depth in the XHMM analysis. LOEUF of each gene is shown as a horizontal line corresponding to its genomic region. In the plot of z score for depth, the red line indicates that of the NDD case with the LOF dnCNV, and the black lines indicate those of 500 randomly selected control individuals.

**
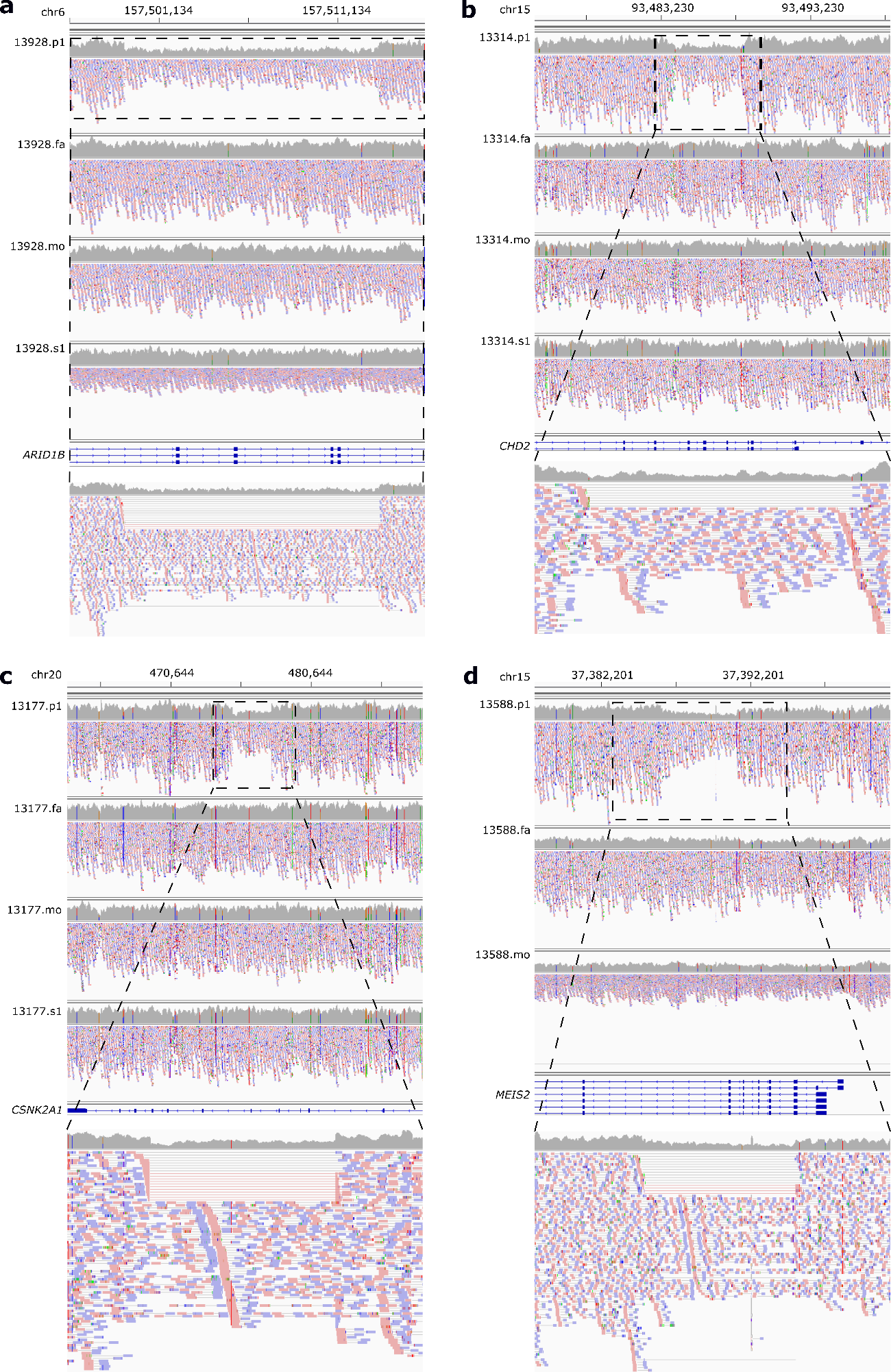
**

**
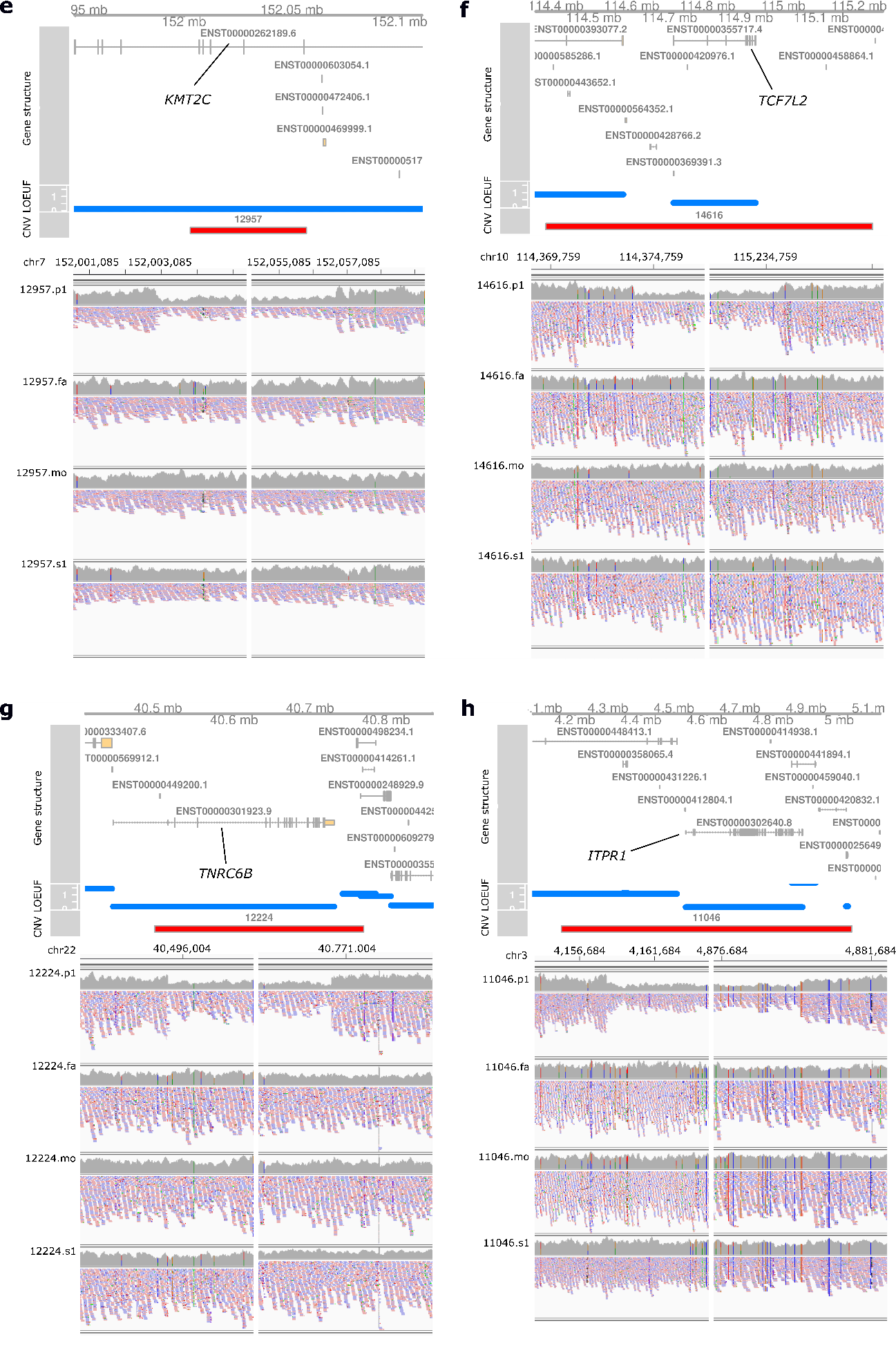
**

**
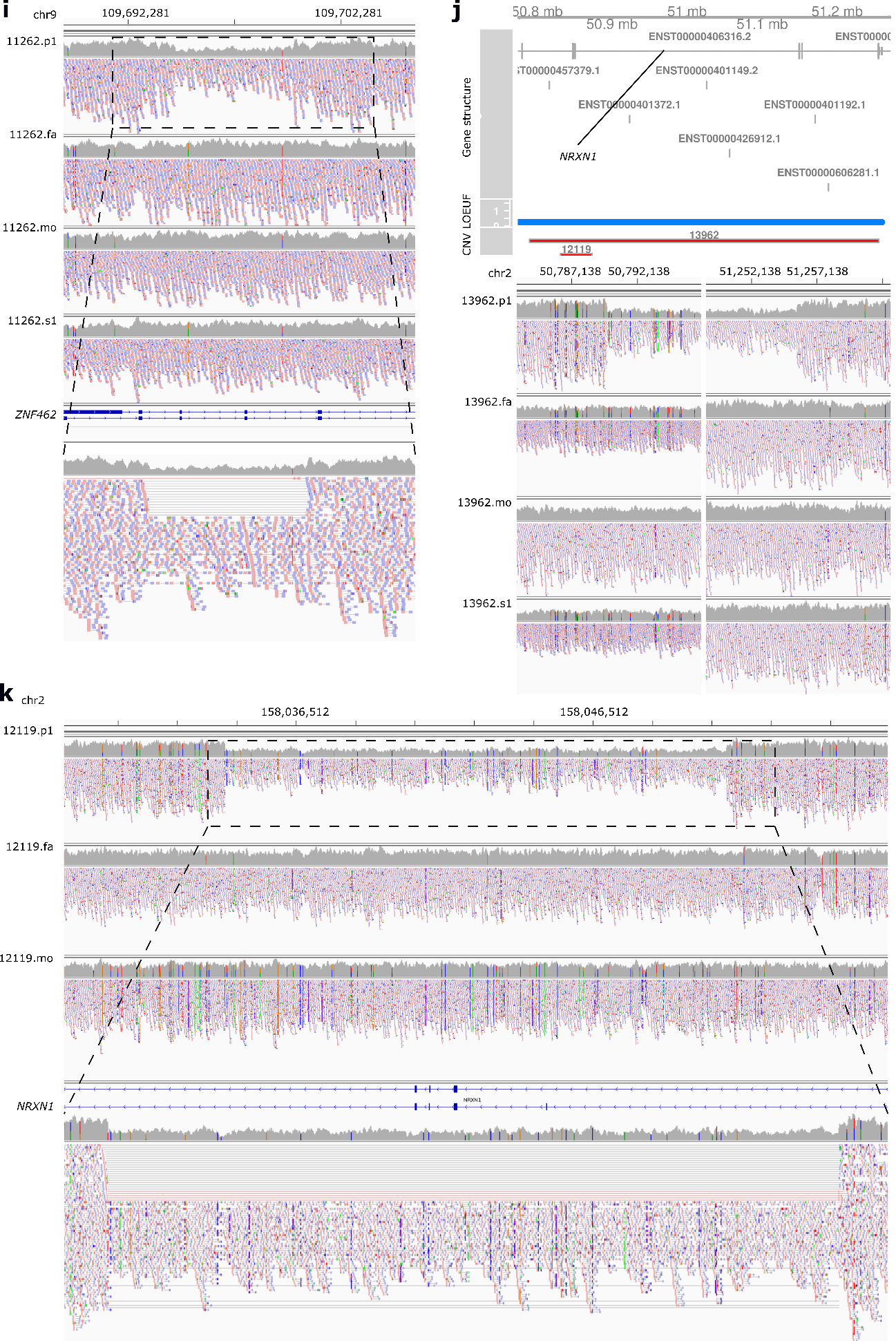
**

**Figure S6. dnCNVs at the 380 DNM-enriched genes in SSC data**

IGV images of dnCNVs as shown in Fig. 2b and 2c. (a–d, i, and k) From the top, coverage and paired-end reads of all family members, exon–intron structures of genes, and magnified images of the coverage and paired-end reads of the affected proband are shown. (e, f, g, h, and j) From the top, the exon–intron structures of the canonical gene transcripts, LOEUF, GenomeSTRiP calls of dnCNVs, and the coverage and paired-end reads of all family members and exon–intron structures of genes are shown. In the magnified images, discordant read pairs, whose read one and read two spanned a dnCNV, are connected with a black line, while split reads, which span a breakpoint, are connected with a red line. (h) Note that the 3’-end (right) breakpoint of the GenomeSTRiP call was different from that in the IGV view and likely incorrect. The 3’-end breakpoint in the IGV view was within the *ITPR* gene region. p1: the affected proband; fa: the father; mo: the mother; s1: the healthy sibling.







**Figure S7. Plots of DNMs at the 52 DNM-enriched candidate genes**

Plots of DNMs in the 52 candidate genes. Exon structures (top, gray), the per-base depth in the WES data in gnomAD (2^nd^ top, light blue), the density (moving average of the variant counts) of missense variants in WES in gnomAD (3^rd^ top, brown), locations of functional domains in Pfam (3^rd^ bottom, color-coded squares by domains), DNMs (2^nd^ bottom, shape- and color-coded as described below), and annotation of the Pfam domains (bottom, color-coded as in the 3^rd^ bottom row) are shown. The ranges of the y-axes of the depth and missense density are the same in all plots as shown below. Depth of WES in gnomAD is shown because it could influence the density of missense variants. DNMs (2^nd^ bottom) are shape- and color-coded as follows: triangle: LOF variants; cross: damaging (> 2.0 Missense badness, PolyPhen-2, and Constraint [MPC]) missense variants; red: DDD31k; green: denovo-db; blue: YCU cohort.


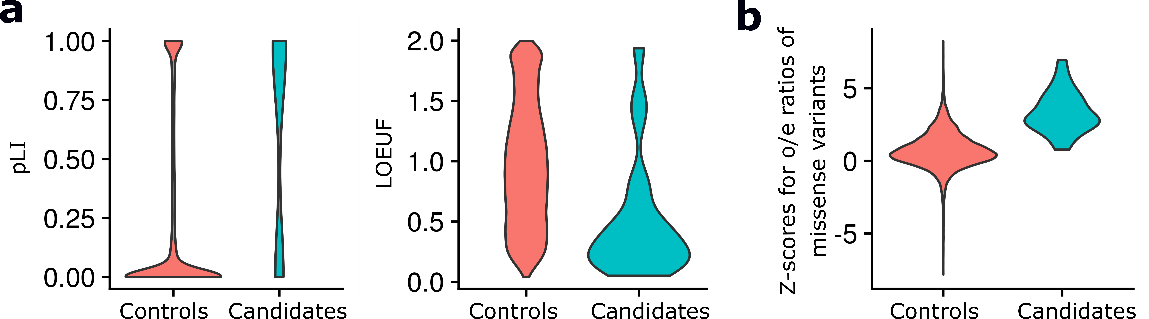


**Figure S8.** **Enrichment of constrained genes in the 52 DNM-enriched candidate genes**

(a) Violin plots of pLI and LOEUF of the 44 candidate genes enriched for LOF DNMs (q-value < 0.05) and 18,286 control genes. As control genes, we used ones not listed as autosomal dominant or X-linked developmental disorder genes in Development Disorder Genotype Phenotype Database (DDG2P), haploinsufficient genes in ClinGen, or the 380 DNM-enriched genes. (b) A violin plot of z-scores of missense variant o/e ratios of the 26 candidate genes enriched for damaging missense (d-MIS) DNMs (q-value < 0.05) and the 18,286 control genes.


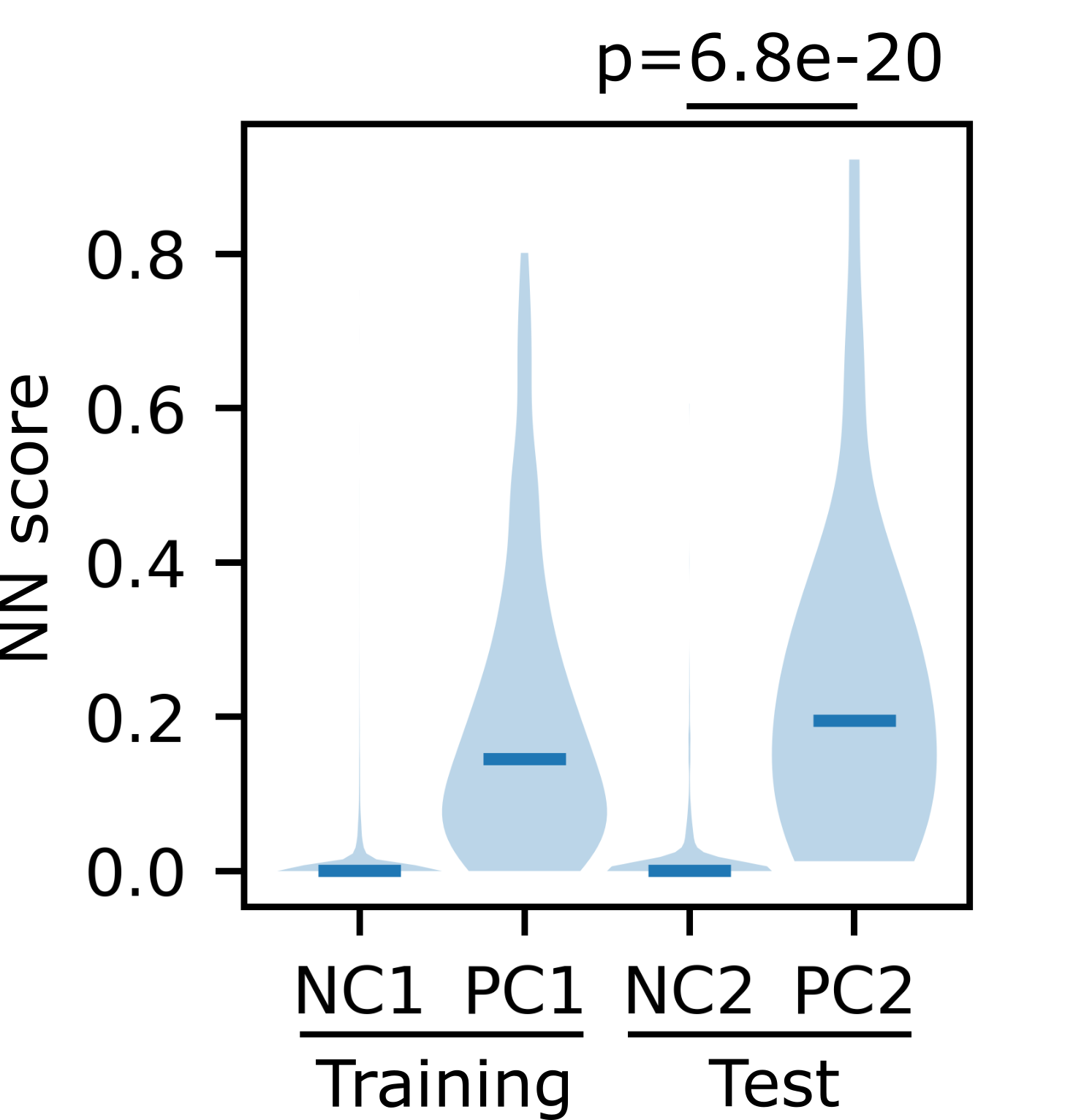


**Figure S9**. **Distributions of neural network model scores in NC1 and PC1 training and NC2 and PC2 test gene sets**

The violin plots of neural network model score distributions are shown. The model was trained on NC1 and PC1. The p-value of a one-tailed Wilcoxon rank-sum test is shown above.

**References**

1. Thorvaldsdóttir H, Robinson JT, Mesirov JP. Integrative Genomics Viewer (IGV): high-performance genomics data visualization and exploration. Briefings in bioinformatics. 2013;14(2):178-92.

2. Hamanaka K, Miyatake S, Zerem A, Lev D, Blumkin L, Yokochi K, et al. Expanding the phenotype of IBA57 mutations: related leukodystrophy can remain asymptomatic. Journal of human genetics. 2018.

3. McKenna A, Hanna M, Banks E, Sivachenko A, Cibulskis K, Kernytsky A, et al. The Genome Analysis Toolkit: a MapReduce framework for analyzing next-generation DNA sequencing data. Genome research. 2010;20(9):1297-303.

4. DePristo MA, Banks E, Poplin R, Garimella KV, Maguire JR, Hartl C, et al. A framework for variation discovery and genotyping using next-generation DNA sequencing data. Nature genetics. 2011;43(5):491-8.

5. Cingolani P, Platts A, Wang le L, Coon M, Nguyen T, Wang L, et al. A program for annotating and predicting the effects of single nucleotide polymorphisms, SnpEff: SNPs in the genome of Drosophila melanogaster strain w1118; iso-2; iso-3. Fly. 2012;6(2):80-92.

6. Hamanaka K, Takata A, Uchiyama Y, Miyatake S, Miyake N, Mitsuhashi S, et al. MYRF haploinsufficiency causes 46,XY and 46,XX disorders of sex development: bioinformatics consideration. Human molecular genetics. 2019;28(14):2319-29.

7. Hamanaka K, Imagawa E, Koshimizu E, Miyatake S, Tohyama J, Yamagata T, et al. De Novo Truncating Variants in the Last Exon of SEMA6B Cause Progressive Myoclonic Epilepsy. American journal of human genetics. 2020;106(4):549-58.

8. Wei Q, Zhan X, Zhong X, Liu Y, Han Y, Chen W, et al. A Bayesian framework for de novo mutation calling in parents-offspring trios. Bioinformatics (Oxford, England). 2015;31(9):1375-81.

9. Liu Y, Li B, Tan R, Zhu X, Wang Y. A gradient-boosting approach for filtering de novo mutations in parent-offspring trios. Bioinformatics (Oxford, England). 2014;30(13):1830-6.

10. Ramu A, Noordam MJ, Schwartz RS, Wuster A, Hurles ME, Cartwright RA, et al. DeNovoGear: de novo indel and point mutation discovery and phasing. Nature methods. 2013;10(10):985-7.

11. Miyatake S, Koshimizu E, Fujita A, Fukai R, Imagawa E, Ohba C, et al. Detecting copy-number variations in whole-exome sequencing data using the eXome Hidden Markov Model: an 'exome-first' approach. Journal of human genetics. 2015;60(4):175-82.

12. Fromer M, Moran JL, Chambert K, Banks E, Bergen SE, Ruderfer DM, et al. Discovery and statistical genotyping of copy-number variation from whole-exome sequencing depth. American journal of human genetics. 2012;91(4):597-607.

13. Fromer M, Purcell SM. Using XHMM Software to Detect Copy Number Variation in Whole-Exome Sequencing Data. Current protocols in human genetics. 2014;81:7.23.1-1.

14. Fitzgerald TW, Gerety SS, Jones WD, van Kogelenberg M, King DA, McRae J, et al. Large-scale discovery of novel genetic causes of developmental disorders. Nature. 2015;519(7542):223-8.

15. Werling DM, Brand H, An JY, Stone MR, Zhu L, Glessner JT, et al. An analytical framework for whole-genome sequence association studies and its implications for autism spectrum disorder. Nature genetics. 2018;50(5):727-36.

16. Ruderfer DM, Hamamsy T, Lek M, Karczewski KJ, Kavanagh D, Samocha KE, et al. Patterns of genic intolerance of rare copy number variation in 59,898 human exomes. Nat Genet. 2016;48(10):1107-11.
